# Hmong herbal medicine and herbalists in Lao PDR: pharmacopeia and knowledge transmission

**DOI:** 10.1186/s13002-019-0307-2

**Published:** 2019-06-13

**Authors:** Jean Marc Dubost, Chiobouaphong Phakeovilay, Chithdavone Her, Audrey Bochaton, Elizabeth Elliott, Eric Deharo, Mouachan Xayvue, Somsanith Bouamanivong, Geneviève Bourdy

**Affiliations:** 1 UMR208, PALOC, IRD, MNHN, Sorbonne Université, Paris, France; 2 Faculty of Pharmacy, Mahosot Road, PO. Box 7444, Vientiane, Lao People’s Democratic Republic; 3 UMR 7533 Ladyss, Université Paris Nanterre, Department of Geography, Nanterre, France; 4 University College London-UCL, 14 Taviton St, Kings Cross, London, WC1H 0BW UK; 5 UMR 152 Pharmadev, Université de Toulouse, IRD, UPS, Toulouse, 31400 France; 6 Institute of Traditional Medicine, Phonepapao village, Sisattanack district, Vientiane, Lao People’s Democratic Republic; 7 Biotechnology and Ecology Institute, Ministry of Science and Technology, Po Box 2279, Vientiane, Lao People’s Democratic Republic

**Keywords:** Hmong, Medicinal plant, Lao PDR, Pharmacopeia, Traditional medicine, TEK, Knowledge transmission

## Abstract

**Background:**

In Lao PDR, the Hmong ethnic group has extensive knowledge about the use of medicinal plants. However, despite the importance of the Hmong pharmacopeia as a primary health care resource, no study has been undertaken to thoroughly document medicinal plant knowledge and its transmission. Objectives of this study are (i) to describe and characterize Hmong pharmacopeia, and (ii) to understand how medicinal plant knowledge is transmitted and spread among Hmong in Lao PDR, in order to assess whether this knowledge base is under threat.

**Methods:**

In order to describe Hmong pharmacopeia, a total of 14 interlocutors were interviewed in three provinces (Bokeo, Xieng Khouang, and Vientiane), using “walk in the wood” methodology. To gain insight about knowledge transmission, semi-structured interviews were conducted with 28 people. Twenty of them were herbalists. Data analysis was performed using univariate analysis for the description of the pharmacopeia. Medicinal plant knowledge consistency was assessed through use and plant name overlapping. Answers to the semi-structured interview on knowledge transmission were analyzed qualitatively.

**Results:**

Three hundred thirty-three different medicinal species were collected. The majority of uses attributed to plants were gastrointestinal conditions (22% of total use reports), gynecological conditions and sexually transmitted disease (12%), skin affections (8%), kidney and bladder problems (5%), physical traumas (5%), and aphrodisiac (or male tonics; 5%). Use convergences are more marked in the gynecological sphere, but there is a strong heterogeneity in practices and knowledge. Medicinal plant knowledge transmission is oral, gained from direct experience since childhood, matrilineal, and kept strictly within the family lineage. Apparent limited consensus on uses might stem from the method of knowledge transmission and to the economic value given to medicinal plants.

**Discussion:**

Use pattern of species from the Hmong pharmacopeia does not appear to be strikingly different from the national Lao pharmacopeia. Differences may lie in the methods and reasons for knowledge transmission. It can be proposed that the economic value given to plants helps in keeping the knowledge alive, and encourages its transmission.

**Conclusion:**

Hmong traditional medicine is constantly evolving in a dynamic process and aims to respond to health problems faced by the local population. Herbalists appear as health fully fledged actors and should be recognized and valued as such.

**Electronic supplementary material:**

The online version of this article (10.1186/s13002-019-0307-2) contains supplementary material, which is available to authorized users.

## Introduction

### Hmong population in Lao PDR

Laos is a multi-ethnic country. Tai populations, of which the lowland Lao constitutes a majority in Laos, have been colonizing the lowlands since the beginning of the thirteenth century. Ethnic minority groups include Mon-Khmer speaking groups (a branch of the Austro-Asiatic linguistic family) such as Khmer, Kantu, Talieng, Ta-Oy groups in the south, and Lamet and Khmu in the north. The other highland populations of Laos speak Tibeto-Burmese and Miao-Yao languages, and include Akha, Hmong, Yao, and Lahu.

Hmong differ very much from the Tai/Lao, the Vietnamese, the indigenous Mon-Khmer population of Laos, and the other Tibeto-Burman hill tribes of northern Laos and northern Thailand. They are a particularly clearly identifiable group, who refer to themselves customarily as Hmong and speak dialects of the western or Chuanqiandian branch of Miao language in the Miao-Yao language family, one of the three main branches of this Miao language [1]. Most authors argue that the Miao/Hmong originated from Southern China, based on folk stories, linguistic, and cultural features they have developed or share with the Han Chinese [2, 3].

Hmong people started to migrate from their original homeland in the mid-nineteenth century as a result of oppression and conflicts with the Chinese. In less than 50 years, groups of Hmong people spread to the mountainous regions of northern Vietnam, northern Thailand, and northern Laos where they are presently found. In the 1970s, due to political conflicts, more than 100,000 fled Laos. The path of exile took them to Thailand, then to the USA, France, Australia, Canada, and Argentina [4].

It is now estimated that the actual number of Hmong speakers around the world is between 4 and 4.5 million. There are approximately 3 million Hmong living in southern China (Yunnan province), 350,000 in northern Vietnam, and 100,000 in northern Thailand. In Laos, they are estimated to be about 230,000, making up about 5% of the total population.

The total Miao population in China in 2000 was estimated at 9,200,000, of whom 3,100,000 are Hmong. The figure for the latter increases to 4.5 million world-wide if we add the following: 787,000 in Vietnam; 460,000 in Laos; 120,000 in Thailand; 2000–3000 in Myanmar; 200,000 in the USA; 15,000 in France; 2000 in Australia; 1400 in Canada; 300 in Argentina; and 110 in Germany [5]. However, these data should be interpreted with caution, as they are almost 15 years old now, and also because there is a debate about the percentage of Miao people that should be defined as Hmong [6].

Hmong are customarily divided into two major linguistic and cultural divisions, referred as the blue (or green) Hmong (*Hmoog Ntsaub*) and the white Hmong (*Hmoob Dawb*), according to the color of women’s traditional dress ornaments. Of greater social importance is the lineage or clan, as it constitutes the basis of Hmong social organization. Eighteen clans have been identified in Thailand and Laos [7]. The clans are patrilineal, and strictly exogamous, meaning that at the time of marriage, a woman leaves her own clan and joins that of her husband. According to Ovesen, a person’s loyalty is always toward other members of his own clan, irrespective of the village or region of residence, and this basic clan solidarity is undiminished even among Hmong living in communities overseas. Indeed, given the migratory lifestyle of the Hmong, clan solidarity is extremely important [4]. Hmong traditionally subsist on swidden cultivation, and it is assumed that on their journey south, Hmong never stayed long enough in a place to develop a sustainable system of shifting cultivation. Most Hmong villages act only as temporary groupings of residential units. Historically, the Hmong were also opium growers, and although opium was once an important cash crop for them, the opium poppy is now grown mostly for medicinal personal use, as it has been the target of various eradication programs [7].

### Hmong traditional medicine and phytotherapy

The lowland Lao pharmacopeia has historically been documented through a number of studies in medical botany. In colonial times, Vidal was a major contributor with more ten publications on the topic [8] together with Petelot [9] then followed by others [10–21]. Although studies have been undertaken in other ethnic groups, there has been little systematic documentation of medicinal plant use among highland groups.

For the Hmong, traditional medicine is practiced as part of a vast cosmology. An outer visible world and an invisible one co-exist and great social and ritualistic importance is given to ancestors [22]. The Hmong practise a combination of animism and ancestor worship. In this belief system, shamanic perceptions stipulate that the person is the host of 12 souls, all essential to life, that must remain in harmony in order to ensure good health [23]. A first type of soul is a permanent resident of the body, while the second type is very unstable by nature. Spiritual illness occurs when one or more of the human souls become separated from the human body or become compromised in some other way, requiring intervention by a shaman. Shivering, difficulty in sleeping, startled responses, loss of appetite, fever, cold hands and feet, hearing and seeing things are psychological indications of having been frightened or suffering from the loss of a soul. The shaman’s role is to restore decaying souls and to fetch those that are wandering and bring them back in the body. Nevertheless, usually Hmong people describe only one soul, the *pli*, which is an essential vital spirit believed to be able to leave the body, causing sudden death [24].

In addition, it is believed that all natural entities such as rocks and bodies of water (such as lakes and ponds) have spirits that may have a neutral, positive, or negative impact on a person’s spiritual well-being. In this system, there is interdependence between people living in the physical or visible world, and the deceased who inhabit the world of ancestors and other spirits. Violation of cultural norms governing relations between these two worlds may result in an ancestral spirit causing harm to the offender, his or her living family members or future descendants of the offender, and be the cause of loss of health [23].

Besides these spiritual causes of diseases, Hmong people believe that some illnesses, particularly those that are less severe and non-life-threatening, may have a biological cause requiring treatment with *tshuaj ntsuab* (herbs) or other organic substances such as dried animal parts [25]. These remedies may be used alone or in combination with spiritual healing. It was also noted that the techniques of acupuncture, massage, cupping, pinching, rubbing, steaming, and burning are also widely used by these ethnic groups and it has been hypothesized that many traditional remedies have been influenced by Chinese traditional medicine [26].

Besides these studies, which have given a glimpse of the intricately linked Hmong medicine and cosmology, there are very few publications documenting Hmong pharmacopeia, especially recent ones. In Thailand, a nurse working in a refugee camp listed the name and uses of 15 plants used by a Hmong herbalist [27]. In 1993, Anderson published a book, “Plants and People from the Golden Triangle” wherein he briefly reported the use of 52 species by Hmong villagers in northern Thailand [28]. In the same country, another author focused on plants used for women’s health and also listed a few species with medicinal uses present in Hmong home gardens in Northern Thailand [29, 30]. Elsewhere, one study focused on the ethnopharmacology of 37 introduced species used by Lao Hmong refugees in Minnesota [31]. In China, a comparison has been made between the traditional knowledge of plant uses by the Hmong and the Li, both groups living around Limu mountain in Hainan island [32].

Finally, in Laos, to the best of our knowledge, only three studies describe very briefly uses of medicinal plants by Hmong communities. In 1970, Vidal and Lemoine were the first to mention the use of species for medicinal purposes [33]**.** Much later, Lundh mentioned the use of six species for women’s problems in villages in northern Laos [34]. A recent study was also undertaken in Luang Prabang in northern Laos [35]. Using quantitative ethnobotany tools, the authors listed 16 species widely used by Hmong herbalists, but did not mention their uses, as this paper addressed conservation issues. Currently, all over Lao PDR, in addition to personal use, medicinal plants are collected in large quantities for sale in markets by Hmong herbalists, mainly women. Nevertheless, to the best of our knowledge, despite the wide use and marketing of medicinal plants by Hmong people in Laos, Hmong knowledge of medicinal plants has never been documented.

Therefore, the objectives of this study are (i) to document the uses of plants by Hmong in three regions of Laos where Hmong communities dwell (two in the northern provinces and one near the capital city Vientiane) in order to describe and characterize Hmong pharmacopeia in Laos, and (ii) to understand the way knowledge of medicinal plants is transmitted and spread among the Hmong. More generally, we hope that this study will draw attention to an important health resource at risk because of overexploitation of natural resources, and will help in the integration of traditional medicines into the Lao national health system as recommended by the WHO (WHO Strategy of Traditional Medicine 2012), which is part of government health policy.

## Methods

### Place of study

**Fig. 1:**
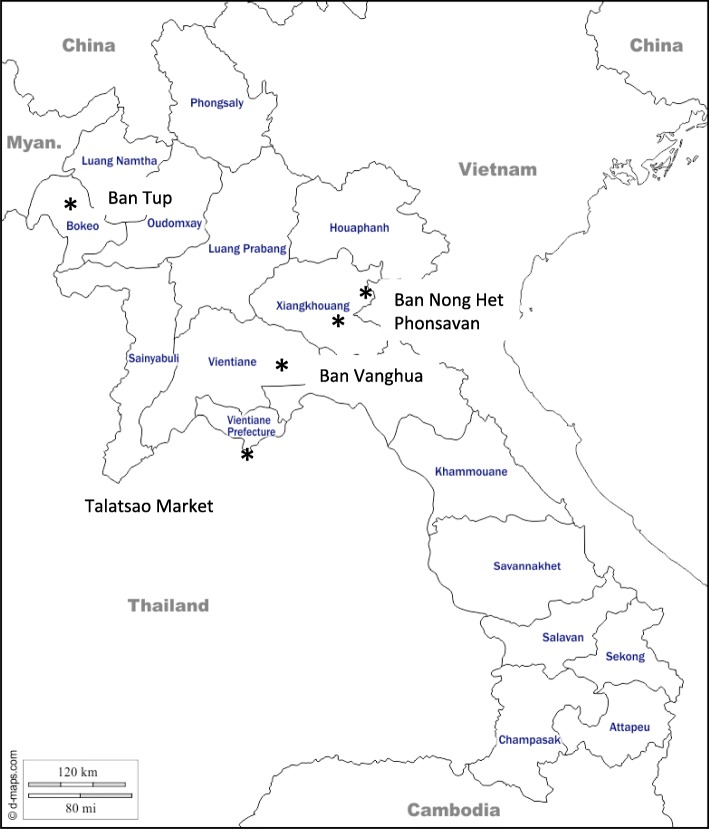
Place of study

This survey took place in three different provinces with Hmong villages (see Fig. 1, place of study): Bokeo and Xieng Kouang Provinces in Northern Laos, and Vientiane Province in central Laos.

In Bokeo province, the study was undertaken in the Hmong village of Ban Tup (Houay Xay district). Ban Tup is a compact village of 49 households, 1.5 h by motorbike from Houay Xay, the nearest urbanized center. Its inhabitants live mainly on swidden agriculture and an ecotourism activity organized in the park (Gibbon Experience Pek district project). Medicinal plant specimens were collected in the forested area composed of upper mixed deciduous forests (primary and secondary growth) surrounding the village, and on the edge of cultivated areas, or along pathways at an elevation ranging between 450 and 800 m.

In Xieng Khouang province, the survey was conducted around Ban Nong Het village (Nongpet district) and in Phonsavan (Pek district) near the Vietnamese border. These districts are populated mainly by members of the Hmong ethnic group. Agriculture is a major component of the family economy together with diversified activities. The market held every Saturday in Ban Nong Het includes a section dedicated to the trade of medicinal plants [36]. In these places, specimens were collected at an elevation ranging from 1400 to 1600 m in pasture areas with scattered bushes and in upper mixed deciduous forests (young and older secondary growth) and also in interlocutors’ gardens.

In Vientiane province, the survey took place in the Hmong village of Ban Vanghua (Hom district) 3-h drive from Vientiane city close to Phu Khao Khuay national park. Specimens were collected at an elevation ranging from 450 to 1000 m in young and older secondary mixed deciduous forests, along creeks banks, in rice field areas, along pathways or in a healer’s garden.

### Ethnobotanical survey

A total of 14 interlocutors were interviewed. Seven live in Bokeo province, four in Xieng Khouang province, and three in Vientiane province. Six were male and eight were female. Age ranged from 16 to 60 (average 43 years old). Among them, eight were traditional healers (seven of them women) with specific knowledge about medicinal plants. Three of the female healers interviewed also sell plants in the market in Vientiane and make a living from it. Other healers worked in their village. Field surveys took place in March 2010 in Bokeo province, July and August 2011 in Xieng Khouang province. Surveys in Vientiane province were undertaken in October 2015 and 2016. In all places, we worked with the help of a translator.

Two methodologies were used in the field.

In the villages, plants were collected during walks in different ecotypes using “walk in the wood” methodology, with the help of interlocutors. Only medicinal plants were collected. For each plant, a herbarium specimen was collected for further identification.

In Vientiane city, a market survey was implemented in the Morning Market where medicinal plant stalls are operated by Hmong women. Names and uses of plants were registered. Plants were then collected in situ in nearby Ban Vanghua village with the help of one of the plant sellers, allowing us to make herbarium vouchers for further identification.

For each specimen collected, relevant details on the plant’s uses, mode of preparation, and administration of remedies were noted and registered. Plant names as well as their meanings were recorded with the help of the translators in the Barney–Smalley Roman transcription of Hmong language, which is widely used by Hmong people living in Laos and Thailand [37].

### Semi-structured interview on knowledge transmission

In order to gain some insight about knowledge transmission and information on traditional medicine practice and plant sales, a semi-structured interview was set up and when possible, addressed to our interlocutors and also extended to other herbalists working in the same area who were willing to participate. A total of 20 women, all knowledgeable in medicinal plants, were interviewed. Informal discussion with five village chiefs and three people, all men, completed these interviews.

### Data analysis

Univariate analysis was used to analyze data relevant with the pharmacopeia description. A medicinal use for a species was defined as a use-report which was recorded [38]. Use-reports were divided into usage categories. Usage categories are groupings of medical conditions that affect a system of the body [32]. Answers to semi-structured interviews on knowledge transmission were analyzed qualitatively. Pharmacopeia consistency was assessed by comparing the use of same species either collected in different places or as described in the literature (use convergence), and also through plant naming.

### Determination of species

All herbarium vouchers were deposited in the National Herbarium of Laos and the species were determined by specialists of the Lao flora: Dr. Vichith Lamxay, Dr. Somsanith Bouamanivong, Mr. Mouachan Xayvue, Dr. Sovanmoly Hul, and Dr. James F. Maxwell (deceased). Only plants determined by a specialist on the basis of herbarium numbers at genus level were included in this study. Synonyms were checked and confirmed through *The Plant List* data base [39].

## Results and discussion

### Pharmacopeia description

#### Plant collection

A total of 378 herbarium samples corresponding to 333 different species (116 in Bokeo province, 91 in Xieng Khouang province, and 157 in Vientiane province) were collected. For species determined only at the genus level, if the genus was the same, they were treated as two different species. Forty-one species were collected more than once in different places of study.

#### Family predominance of medicinal plants

The plants collected belong to 105 families. The most represented families with the highest number of different medicinal species collected were Leguminosae (25 species), Zingiberaceae (23), Rubiaceae (21), Compositae (18), Euphorbiaceae and Poaceae (9), Rutaceae (8) Acanthaceae, Araceae, and Moraceae (7 each), Apiaceae, Asparagaceae, Lauraceae, Myrsinaceae, Orchidaceae, Amaranthaceae, Solanaceae, Sterculiaceae, Verbenaceae, Vitaceae (6 each). In a study performed in Bolikhamxay province among Lao traditional healers from the Lao-Loum ethnic group, it was also found that Leguminosae, Rubiaceae, and Rutaceae had the highest number of species used medicinally [12]. Moreover, the importance of Zingiberaceae in Lao traditional medicine has been earlier highlighted [8], as in Cambodian ethnic groups [40] and in Thailand [41]. In Laos, species of Zingiberaceae (mainly *Kaempferia* and *Zingiber* genus) with a strongly aromatic rhizome or root form part of a group of plants denominated “*wan*.” Cultivated or collected in the wild, they are considered to be very powerful, and some of them can be manipulated to cause death, while others can act as antidotes (*Kaempferia galanga* L., *Zingiber montanum* (J. Koenig) Link ex. A. Drietch). Other uses include treatment of snake bite *Kaempferia elegans* Wall ex Baker, digestive troubles, and fever (*Zingiber zerumbet* Roscoe ex. Smith and *Zingiber montanum*) [8].

In this survey, Zingiberaceae were found to be mainly used for digestive disorders (underground parts of all *Curcuma*, *Kaempferia*), to increase male sexual potency (*Boesenbergia longiflora* (Wall) Kuntze, *Kaempferia parviflora* Wall ex Baker), for neuromuscular problems such as paralysis, neuralgia (*Alpinia oxymitra K*. *Schum*, *K*. *rotunda* L.), for infections of the genital sphere (*Curcuma comosa* Roxb. and *C*. sp.) or of the skin (*Alpinia conchigera* Griff and *A*. *galanga* L. Willd.), while *Amomum* sp. are used for cough or hoarseness.

#### Distribution of plant parts

**Fig. 2:**
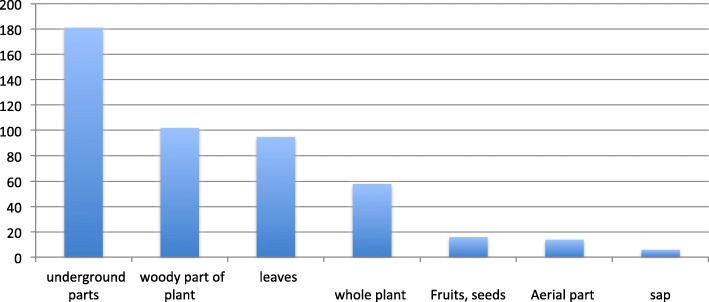
Distribution of plant parts

The underground parts of plants including rhizome, corms, roots, and tuber were by far the most used for their medicinal properties and totaled the highest number of citations (see Fig. 2, Distribution of plant parts). Next most frequent were the woody part of plants (bark, wood, inner wood, and stem), followed by leaves. In 9% of the recipes, different plant parts of the same species are used together. Roots are combined with stems in 13 recipes, and roots are mixed with stems and leaves in 12 recipes. Interestingly, one of our interlocutors, a woman healer selling plants on the market, told us that according to the Hmong tradition, the best plant part for all type of remedies is always the root. However, because of the ongoing scarcity of some medicinal plants now, she felt it necessary to change roots for pieces of stem. This was also pointed out by other interlocutors, reaffirming that roots can be replaced by other plant organs (11 use reports). The importance given to the underground parts of plants as reported here is similar to other studies performed in Laos [12] and more broadly in Asia [42]. For example, in a compilation of traditional Chinese materials, more than one-fourth of the over 400 preparations are derived from roots and/or rhizomes [43].

#### Plant uses

**Fig. 3:**
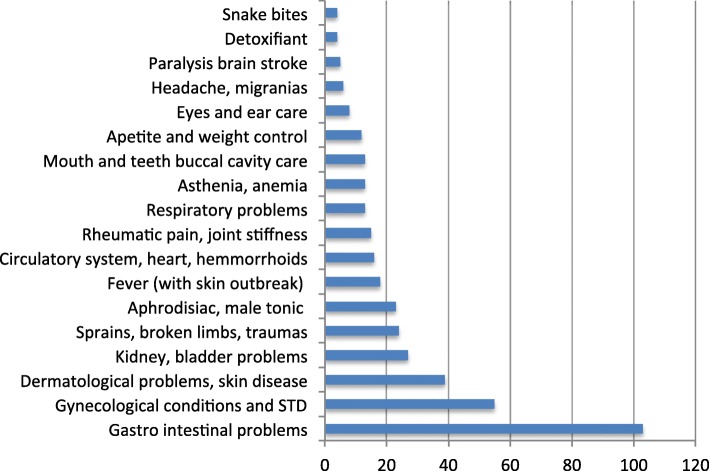
Distribution of use reports

Four hundred sixty-four use reports corresponding to 333 different species have been registered (for a complete overview of medicinal plants uses, see Additional file 1, Hmong medicinal plants use). In the order of importance, the majority of uses attributed to plants concern the gastrointestinal sphere (22%), followed by various gynecological conditions and sexually transmitted disease (12%), skin affections (8%), kidney and bladder problems (5%), physical traumas (5%), and aphrodisiac (or male tonics, 5%) (see Fig. 3, Distribution of use reports). Other uses were of minor importance. It is fair to assume that this distribution is similar to that found in other regions of Laos [12]. We will detail here only the medicinal uses of plants with the highest number of use reports.

#### Gastrointestinal problems

One hundred three uses (including diabetes), corresponding to 96 species were registered, thus making gastrointestinal problems the major health issue treated with plants.

The main indication related to gastrointestinal disorders is stomach pain (29 use reports). Twenty-nine species were found to be useful in case of gastritis with moderate to severe pain, and stomach ulcers. Indeed, gastritis is a very common health problem in Laos, especially among men. According to some interlocutors, this may be due to the habit of drinking too many alcoholic drinks of poor quality. In one recipe, different plant species are combined for more efficiency (i.e. *Hedyotis capitellata* Wall ex G. Don, *Senna alata* (L.) Roxb., *Embelia ribes* Burm.f., *Aphanamixis polystachia* (Wall.) R. Parker). Bleeding ulcers together with acute stomach pain are preferably treated with species from the Iridaceae family (*Eleutherine* spp., *Iris collettii* Hook.f). Four species of Zingiberaceae (*Kaempferia* spp., *Curcuma* sp.) were said to be useful in the case of chronic pain due to an excess of alcohol. In Laos, pills are processed with *Eleutherine subaphylla* Gagnep. bulb, *Curcuma longa* L., *Zingiber montanum* rhizome and sold for gastritis and stomach ulcers [15]. All treatments should be taken internally, and repeatedly over time to cure the problem.

Twelve plant species were reported to be useful in case of liver problems, mainly jaundice, or swollen tough and painful liver, associated with difficulty in breathing. To treat swollen liver, *Aglaonema simplex* (Blume) Blume, *Angiopteris ceracea* Alderw, *Clerodendrum glandulosum* Lindl., *Selliguea* sp., are mashed or finely grated and prepared in poultice form, placed directly in situ, and renewed everyday. Liver problems including hepatocarcinomas are highly prevalent in Lao PDR as well as neighboring countries, and it has been shown that because no effective treatments exist, people turn to traditional medicine and traditional healers [44].

Among all the species listed in this survey, *Oroxylum indicum* (L.) Kurz is a Bignoniaceae species also widely used for liver-related problems in Lao PDR, China, Vietnam, and Cambodia. Its methanolic, ethanolic, and aqueous bark and wood extracts have been shown to have hepatoprotective properties [45] and to contain antiproliferative compounds against the carcinogenic HepG2 cell line [46]. The mushroom *Ganoderma lucidum* (Curtis) P. Karsten growing on decaying woods, together with the small upland orchid *Anoectochilus lylei* Rolfe ex Downie, both cited in this survey, are highly prized ingredients for serious liver problems. Potential pharmacological activities of *G*. *lucidum* have been much sought after in the field of cancer, neurology, and immunomodulation, thus making this fungus widely used in alternative medicine over the world [47]. This high demand generated the necessity for large-scale cultivation [48], but in Lao PDR, this forest fungus is still collected from the wild in large quantities. Now becoming scarce, its retail market price is quite high.

Diabetes is a health condition recognized as a major concern by our interlocutors. It was stated that diabetes can be diagnosed when drops of urine poured on the floor attract ants (because of their sweet taste) and also when the person needs to drink a lot of water. Plants said to be bitter (*Tinospora* crispa (L.) hook. f. Thomson, *Solanum torvum* Sw.), or to possess the five tastes (sweet, sour, salty, bitter, and acrid) such as *Phyllanthus emblica* L. fruits are much favored for this condition. Decoctions of these plants drunk during the day are thought to help to regulate the blood sugar level.

Slow digestion, dyspepsia, bloating after eating, and a swollen stomach are treated with 12 species, among them three *Kaempferia* rhizome, either eaten raw (*K*. *cochinensis* Gagnep.) or prepared in the form of a decoction (*K*. sp. mixed with *K*. *galanga*). For prevention, digestive potency can be enhanced by *Millettia caerulea* Baker and *Dianella ensifolia* (L.) DC. prepared in decoction.

Food poisoning, including eating spoiled food, has been quoted repeatedly as a cause of intestinal pain, stomach inflammation, diarrhea, and vomiting. This condition can be prevented by eating pieces of *Curcuma* sp. rhizome before and after meals (or consuming any suspicious drink). Three Zingberaceae species were said to be very useful to treat that condition (*Costus* sp. *Boesenbergia rotunda* (L.) Mansf., *Alpinia* sp.). *Morus alba* L. trees, cultivated on a large scale in in Lao PDR for the sericiculture industry, are also widely used for digestive problems, and the leaves are frequently used as a herbal tea.

In this survey, Guava (*Psidium guajava* L.) widely used all over the tropics against diarrhea [49] has been cited twice for this use, over ten species.

*Embelia ribes* was said to be useful to expel taenia worms when mixed with other plants (*Hedyotis capitellata*, *Senna alata*, *Aphanamixis polystachia*). Indeed, the use of *E*. *ribes* fruit as antihelmintic is repeatedly quoted in a number of galenic preparations for this purpose in Ayurvedic or Unani pharmacopeias [50]. This use has also been registered among a Lao minority in the north of Lao PDR, where pills are prepared from grinded, decorticated, dried fruits [15].

Finally, the Asclepiacdaecae *Thottea tomentosa* (Blume) Ding Hou, taken internally, helps in reducing gallbladder inflammation and very bitter fruits or bark of *Aphanamixis polystachia* are efficient remedies for severe intestinal inflammation and infection (a condition said to be associated with appendicitis).

#### Dermatological conditions

Different type of skin affections including burns, dermatitis (i.e., eczema and other allergic manifestations), dermatosis, wounds, skin outbreaks (spots, with or without fever) represent 39 use reports concerning treatments with 39 species.

Burns are treated with *Aloe vera* (L.) Burm.f. transparent mucilagenous jelly. In some recipes, freshly picked leaves are wrapped in banana leaves, softened over the fire or directly heated over a flame. These pre-heated leaves are then applied as a lukewarm poultice on the skin. The same type of treatments is used to treat dermatitis, skin allergy, skin itching, or rash in adults and small children. Severe dermatitis due to contact with hairy caterpillars or Anacardiaceae tree sap are treated in the same way.

In case of severe skin damage with swelling, liquid exudation, frequently renewed topical application of poultices is recommended together with internally administered decoctions of *Reynoutria japonica* Houtt., *Croton* cf. *dongnaiensis* Pierre & Gagnep.

Poultices of freshly crushed or sliced plant parts applied directly on skin are used in case of dermatosis (scabies, ringworm) or for other skin infections, i.e., furunculosis or sores that do not heal spontaneously. However, regarding *Alpinia conchigera* Griff., it is advised to apply only briefly a small amount of the crushed rhizome, as long-lasting contact might damage the skin. It should be noted that this Zingiberaceae species together with *Alpinia galanga* (L.) Willd., also used for dermatosis, have been shown to possess antimicrobial activities when used as essential oils [51, 52]. Both *Senna alata* and *Centella asiatica* (L.) Urb are widely used pan-tropical species for skin problems, displaying strong activity against various dermatological infectious pathogens [53, 54].

Quite a large number of plant species [9] have been mentioned as useful for large and profound wounds, perhaps because agricultural and forestry practices place the person at risk of badly cutting himself. Plants used for wounds are said to have hemostatic properties (*Ageratum conyzoides* (L.) L., *Chromolaena odorata* (L.) R.M. King & H. Rob., *Leea indica* (Burm.) f. Merr., *Mahonia napaulensis* DC., *Zephyranthes* sp.), antiseptic effects (*Amischotolype mollissima* (Blume) Hassk, *Ludwigia* sp.), or to facilitate the cicatrization process (*Smilax ovalifolia* Roxb. ex D. Don). *Chromolaena odorata* is a native from Central America. In Lao, it is named “French herb” and people say that this plant was introduced by the French during colonial times. In all places where this tall herb grows, its freshly crushed leaves have a strong reputation as anti-hemorrhagic and to stop bleeding from wounds [55, 56]. Indeed, some interviewees stated that the leaves of this species, together with *Zephyranthes* sp. (whole plant), are among the most efficient for this use. Finally, a treatment for skin ulcers is made with the underground part of two Araceae species (*Colocasia fallax* Schott and *Homalomena* sp.) or with the large, enlarged base of a *Nephrolepis undulata* (Afzel ex Sw.) J. Sm. fern frond, ground into a paste.

#### Kidney and urinary tract problems

Twenty-six species, corresponding to 27 use reports, have been quoted for their effects on the kidney and urinary tract. Twelve species were said to be employed specifically to treat kidney stones. Health problems related to urolithiasis appear to be frequent among the whole male population of Laos. According to some authors, this high number of cases could be explained by the high quantity of freshly picked leaves eaten everyday. Indeed, it has been demonstrated that in Lao PDR, high consumption of green leaves is correlated with a significant higher urinary excretion of oxalates and cases of urolithiasis [57]. Plants said to be useful in the treatment of kidney stones are said to also have diuretic properties. Four Poaceae species were reported for use for this indication: *Cynodon dactylon* (L.) Pers., *Coix lacryma-jobi* L. seeds (cited twice), *Chrysopogon aciculatus* (Retz.) Trin., and *Lophatherum gracile* Brongn. All these species, belonging to Asian pharmacopeias, have been known for a long time to have an effect on the urinary tract and their use has been scientifically validated [58–61]. Frequent miction, which may result from prostatic problems, is treated with five species. All remedies are taken internally throughout the day in the form of drinking water “to clean the kidneys” (*Uncaria macrophylla* Wall).

#### Sprains, broken bones, and trauma

Twenty-four use reports were recorded for treatment of sprains, fractured bones, wounded or torn ligaments, tendinitis, bruises, or any other type of physical trauma, making up 23 recipes. More than half of them (14) are for broken limbs. Indeed, trauma is said to occur frequently, and the person affected is generally treated at home, wish the help of some easily located plants. Two species (*Sambucus javanica* Blume and *Bridelia stipularis* (L.) Blume) were cited for this same use in two different places, thus displaying an interesting use convergence. Plants are always used freshly collected, mashed, or crushed and applied as a poultice, which is regularly renewed. Edema following sprains or tendonitis can be reduced by an internal administration of *Ziziphus oenopolia* (L.) Mill. or *Senna hirsuta* (L.) H.S. Irwin & Barneby root decoction. Also, in case of bruises or suspicion of a trauma that would have caused internal bleeding, a concentrated decoction of the bulb of an Iridaceae species *Sisyrinchium palmifolium* L. or the strongly scented Rutaceae *Toddalia asiatica* (L.) Lam. root are said to have quick, strong anti-hemorrhagic actions.

#### Aphrodisiac, male tonic

Quite a high number of use reports (22) were noted for this indication, which reflects the importance given to aphrodisiacs, widely reported in Southeast Asian pharmacopeias. Indeed, the use of plants to increase male sexual performance is quite common in Asia, although most of the time it is almost impossible to ascertain which ingredients are in the countless preparations sold for that purpose [62].

In this survey, species were generally described as restorative, or “tonics,” giving strength to the body in general, invigorating men at any age, promoting sexual endurance, and increasing male sexual performance. *Ardisia* spp. were cited twice for this use, while two Zingiberaceae species (*Boesenbergia rotunda* and *Kaempferia parviflora* (underground parts) which are cultivated on a large scale) are highly prized. Indeed, recently *K*. *parviflora*, so-called black ginger, gained much popularity and has been formulated in many preparations aiming to increase physical and work capacity. *Boesenbergia rotunda* (Chinese ginger or finger root) is a culinary spice recently studied for its aphrodisiac properties [63–66].

Aphrodisiac power is generally ascribed to roots (or underground parts of plants) macerated in alcohol. In a few recipes, roots are mixed with piece of stem or bark from the same species. Alternatively, in some recipes, crushed root is added to chicken or pork broth, or a pig stomach is stuffed with the selected plants, cooked and eaten. According to one of our interlocutors, the most active species for this use is an Acanthaceae, *Phaulopsis dorsiflora* (Retz.) Santapau.

#### Women’s problems, gynecological conditions, and sexually transmitted diseases

Fifty-five use reports for different types of gynecological conditions were reported, corresponding to 54 species. Indeed, seven of our interlocutors were women, and one of them specified that she gained medicinal plant knowledge from her mother in-law who was working as a midwife.

Use of medicinal plants for female disorders and practices linked with reproduction within the Hmong group have been studied by Lundh (2017) working in Luang Namtha province in Northern Laos [34], who reported the use of six species. Srithi (2012) and Anderson (1993) worked in a Northern Thai Hmong village, and reported the use of 79 and 12 species respectively, highlighting the importance of medicinal plants for female disorders [30, 28]. Detailed anthropological studies focused on the Hmong female reproductive life cycle also show the importance of medicinal plant use [67].

In this survey, one species was designated as a possible abortive (*Thunbergia grandifora* (Roxb. ex Rottl.) Roxb., root) and three species (*Blumea balsamifera* (L.) DC., *Pseuderanthemum latifolium* B. Hansen, *Melastoma malabathricum* L., all roots) were said to have contraceptive properties if administered repeatedly as a decoction during women’s menstruations. Interestingly, in northern Laos, Lundh stated that a decoction of “Jaj to,” *Melastoma* sp. root, was only to be drunk by elderly women who did not want any more children as it could cause permanent infertility [34]. The overall low number of plants found to decrease fertility in our study is consistent with the reports of other authors. This can be explained by the fact that, for Hmong women, power and value are located in the realm of reproduction, so they would, therefore, be reluctant to use any form of abortifacient or birth prevention [34, 67, 68].

In this survey, three species belonging to the genus *Clerodendrum*, *Bryophyllum*, *Galium* were used for miscarriage prevention. According to Hmong women, miscarriage is due to a weakness of the fetus, which manifests as nausea during the first months of the pregnancy, and is believed to be a sign of an unwanted abortion. Symonds noted that these spontaneous abortions are particularly feared because for the Hmong, miscarriage is not only the physiological failure of a pregnant woman to bear a child, but is also considered a failure to extend the clan and lineage, and it has profound meaning related to the spirit and the soul of the unborn. Thus, every attempt is made by Hmong pregnant women to avoid miscarriage. This is why herbalists who know plants that can prevent unwanted abortion are highly respected and often consulted [67].

Fourteen use reports were recorded for the postpartum period. In Hmong culture, as well as in the Lao culture (and more broadly in South East Asia), the time following birth is recognized to be at high risk for the parturient. When the mother gives birth, she is considered to be in an unbalanced “cold” state, the same as when she has her period, making her highly vulnerable. Therefore, it is very important for the mother and child to be kept warm during the month following the birth, especially for the first 3 days. Still, Hmong women do not follow the tradition of lying by the fire (mother roasting) a practice commonly found in lowland Lao and some other ethnic groups, and which is widespread in Southeast Asia [67, 69]. There is also the need for the parturient to stick to strong dietary restrictions, as it is said that eating “wrongly” might cause serious health issues for the woman. Cold water and cold food (i.e., fruits and vegetables) are prohibited, because they are said to damage the person’s blood flow. It is explained that if the blood of a woman is unable to flow freely out of the body, this would cause various sicknesses in old age. Therefore, the main dish for women who have recently given birth consists of chicken boiled with special herbs. It is commonly said in Southeast Asian cultures that chicken is a heat-building and strengthening food. Herbs added to the broth are said to clean the uterus and remove any stale blood, therefore restoring it to perfect condition for the next conception and birth.

In this survey, all the plants administered to women during this postpartum period were said to help in avoiding afterbirth complications (hemorrhages, fever), to give strength to the mother, clean the uterus, and avoid further health problems. The large majority of the species administered during post-partum, including the galactagogues, were prepared in form of chicken broth, using soft parts of plants. Among all plants cited, *Smilax* spp. tuber was mentioned twice [70]. *Thunbergia grandiflora*, a plant already mentioned for its abortive properties, was also cited as useful post-partum because of its strong action on the womb. *Sambucus javanica* was also repeatedly mentioned as a fertility enhancer [28, 33].

Leucorrhea and vaginal discharge with pelvic pain and persistent sensation of itchiness in the sexual organs are recognized as serious conditions, and the same for gonorrhea in men. These indications represent 26 use reports, corresponding to 21 remedies and 24 species, hence highlighting the importance of treating such conditions. *Microglossa pyrifolia* (Lam.) Kuntze and *Urena lobata* L. taken internally or as a local wash have been cited twice for these uses in two different places. It should be noted that *U*. *lobata* is also used in Northern Thailand, to avoid miscarriage, which makes it a species of great importance for women’s health [28]. The ethnogenus *Mussaenda* spp. (*M pubescens* Dryand. and *M*. *sanderiana* Ridl. can be used indifferently) is another important species for these uses. It has been cited three times in this study, thus displaying a strong use convergence and also in Hmong villages in northern Laos [34]. Finally, for these types of infectious conditions, rhizomes from *Curcuma* species (*C*. *zanthorrhiza* Roxb., *C*. sp., *C*. *comosa*) have been much cited for use prepared in decoction and taken internally.

### Pharmacopeia consistency

We tried to broadly assess the homogeneity/heterogeneity of the Hmong pharmacopeia, by making a comparison between the names and uses of the same botanically determined species, collected in different locations. Our data were compared to other authors (in Laos: [33–35] and in Thailand: [27–30]). It should be noted that Whitney (2014) working in Luang Prabang did not specify any medicinal plant uses [35], and that Anderson (1993) and Delang (2007) did not mention any Hmong vernacular name in their study [28, 70].

#### Use convergence

In this survey, 41 species were collected in duplicate or triplicate in different places of study. Thirty-six percent (14) of them were used for a similar medicinal indication. In addition, use convergence was noted for some close-by taxons, collected in different places. This was the case for three *Ardisia* spp. (male tonic). *Clausena excavata* Burm.f. and *C*. *lenis* Drake (fever), *Clerodendrum glandulosum* Lindl. and *C*. *schmidtii* C.B. Clarke (gynecological purposes), *Dioscorea cirrhosa* Lour. and *D*. *hispida* Dennst. (diarrhea, dysentery), *Eleutherine* sp., *Eleutherine subaphylla* (excessive bleeding), *Embelia parviflora*, *E*. *sessiliflora* Kurz, (gastrointestinal disorders), *Eriocaulon buergerianum* Körn, *E*. *sexangulare* L. (eyesight), *Ficus hispida* L.f., *Ficus sp*. (rheumatic pain, paralysis), *Helicteres isora* L., *H*. *hirsuta* Lour. (gastrointestinal problems), *Mussaenda pubescens*, and *M*. *sanderiana* (leucorrhoea), *Smilax* spp. (used as tonic, postpartum, gastrointestinal disorders). The highest number of use convergences [8] noted by far is in the gynecological sphere.

The medicinal use of 28 species collected in this study were also reported in other studies [27–30, 33, 34, 70]. Less than of half of them (15 or 44%) presented use convergence with our data. Again, we observed the highest number of use convergence for species used for gynecological purposes (nine citations over 15).

In overall, these data suggest a general trend characterized by low overlapping of uses, with the notable exception for plants used for gynecological issue. Still, these results should be considered as preliminary. Firstly, because a medicinal species may have different uses not necessarily recorded at the time of the interview. Secondly, as half of our interlocutors were women and healers, one can make the hypothesis that more plants used for women would be known by them, as highlighted by Shrithi (2012) in her publication focusing on this type of knowledge [30].

#### Plant naming

Although the Lao language is the usual language used in Laos for inter-ethnic communication, our Hmong interlocutors, when asked, could very seldom provide a Lao name for the plants they were showing us, suggesting that the plant knowledge is mostly inherited from their own culture. Nevertheless, a few exogenous inputs resulting from contact with other ethnic groups can be highlighted, through the names of a few plant species [33]. In this study, only two species were named after the source from which the knowledge has been drawn. *Celosia argentea* L. in Ban Tup is named *paj maj* meaning “flower Lamet,” as the use of this plant has been indicated by Lamet people, and *Streblus* sp. has a hybrid Hmong-Lao name: *tshuaj maknao hav zoov*, where *makna*o is the Lao name for lemon.

Hmong language is monosyllabic, which means that the morphemes coincide with the syllables, or in other words that each syllable of a noun is significant by itself [37]. Vidal and Lemoine (1970) provided some insights on Hmong ethnotaxonomy [33]. Plant denomination in Hmong follows the same general pattern as in Thai or Lao language where names are formed by one generic word followed by specificative(s) term(s). According to these authors, the generic term refers to the plant biological type (*hmab*: vine, *pos*: thorny, *ntoo*: tree, *nroj*: herb or treelet, *suab*: fern).

In this survey, 386 different names were recorded. Among these, 60% (236 names) include a generic term, which display a much greater variability than previously noted by Vidal, as they referred to a specific part of plant (*nblooj*: leaf, *paj*: flower, *txiv*: fruit, *qhaus*: rhizome, *cag*: root, *pos*: thorn), the medicinal use of the plant (*tshuaj*: medicine), its food categories (*zaub*: leaf vegetable, *qos*: edible tubers), or its cultivated origin (*vaj*: from garden). Also, it was noted that this generic term can be facultative: in our corpus *Aphaenandra uniflora* (Wall. Ex G. Don) Bremek, *Chromolaena odorata*, *Pothos scandens* L., *Solanum spirale* Roxb., *Psidium guajava* were named by different people, one giving the vernacular name with a generic syllable, the others without.

Specific terms are composed from one up to four significant syllables, and refer to a wide range of different meanings. These include medicinal indications (*Smilax ovalifolia* and *Spermacoce remota* Lam. names are literally translated as “(vine) medicine apply ligament” and “good care hurt tooth”, which reflect their use), a morphological specificity of the plant (*Leea rubra* Blume ex. Sprengl. is “(root) big”, *Embelia ribes:* “(liane) 30 leaflets leaf”), a metaphor: *Schefflera elliptica* (Blume) Harms is “hand forest spirit” (because the petiol has the shape of a small hand gripping the stem), and *Uncaria macrophylla* “[thorn] horn sheep green” for the noticeable shape of the leaf is an organoleptic characteristic of the plant (*Clausena lenis* is named “[tree] dog pee”, for the smell of this species).

From the 392 different vernacular names recorded in this study, 20 appear repeatedly. Only six of them refer to the same species, thus showing a lack of overlap between Hmong names and botanically defined species.

Hmong plant name comparison (comparison was performed on the specificative part of the name) was also performed on the 41 species collected more than once in the frame of this study and on the 28 species collected in this study and also mentioned by other authors [27, 29, 30, 33–35].

In both cases, the percentage of names overlapping for the same species was small, as for a given species less than half of the names were similar (44% when comparing between species collected in this study and 46% if comparing with the literature). Moreover, poor plant name consistency was also observed within the same areas: in Ban Tup, a compact village of 49 households, ten species were collected in duplicates, of which seven bear different names; within Nongpet district, four species collected in duplicates were shown to us under different names.

In general, a first examination of both uses and plant name overlapping seems to indicate a strong heterogeneity in practices and knowledge. The same observations were made by Anderson (1983) when working in hill tribes of northern Thailand, stating that “In fact plant use, both within a tribe and amongst other hill tribes is surprisingly inconsistent. Two evident exceptions are the introduced plant Eupatorium odoratum (anti-haemorrhagic) and Aloe vera (burns)” (p. 29). This affirmation was corroborated latter by Pake (1986): “Hmong herbalists do not share their knowledge of plants with one another and, therefore, do not use a given plant for the same purpose or in the same way if treating some ailments [27].” In our opinion, the formation and settlement pattern of Hmong villages may partly account for this heterogeneity in plant names and associated knowledge. Still, it would be interesting to design a comparative study, in order to draw definitive conclusions about this apparent scattering of knowledge.

### Knowledge transmission

All of the women interviewed defined themselves as *Kws tshuaj* (herbal healers) in opposition to *tu txiv neeb* (the shaman who deals with problems originating in soul disorders). They give advice about treatment, sell plants, and provide instructions for their use. Plants are delivered according to the symptomatology described by the customers, or the pathology diagnosed by the hospital or the general practitioner. When asked about their source of knowledge, all the women herbalists interviewed stated that they firstly learned medicinal plant uses from their mother (or grandmother when the mother passed away), who was also a healer, well trained in medicinal plant uses, or from a midwife. Medicinal plant use was generally approached by direct experience, through healing sessions or births, and during walks in the forest or in gardens when the young girl was accompanying her mother.

Once married, some of the women also learnt from their mothers-in-laws (if they were knowledgeable about medicinal plants use) disclosing freely their recipes to their daughters-in-law. Besides these two main sources of knowledge, some women told us that they bought one or two recipes for specific ailments from other healers, but this was anecdotal. It was also stated that recipes disclosed by other healers are always very expensive, and it was noted that payment must be accompanied by a special ceremony to get the agreement of medicinal spirits, in order to avoid health misfortunes [71]. Dreaming about medicinal plants was another type of transmission mentioned, but this was quoted only in two cases as extra information to already transmitted knowledge, and thus seems to be a minor source of information. In turn, all the women interviewed mentioned that they were committed to passing on their knowledge to one of their daughters or, if they did not have a daughter, to a very close niece.

In this survey, we interviewed six men about medicinal plant uses while collecting plants in the wild with them. Only one of them was a renowned herbalist, who had specialized knowledge on medicinal plants that he gained from direct transmission from another herbalist, and was also a shaman. The five other men knew some medicinal plant uses, but did not present themselves as specialists or professional herbalists. They did have a basic knowledge they got from their domestic surroundings, watching other people using plants in daily life, or that they have been using themselves in various life occasions, but would not make a living from this. Therefore, it appears that there are two types of knowledge: a specialized, monetizable type, alongside popular knowledge that is open to all. According to in-depth interviews with female herbalists, the transmission is usually matrilineal, but this does not exclude the fact that men too can have access to this specialized knowledge, either through an initiation, from master to disciple, or by buying this knowledge.

These data are congruent with what Lemoine stated in his doctoral thesis a study which took place in a small village in Sayabouri province and lasted 2 years (from November 1964 to January 1967, almost 50 years ago) [5]. “Medical art is open to all men and women but is nevertheless dominated by experts, the herbalists *kws tshuaj*, who combine the knowledge of medicinal plants with that of certain magical operations whose secret they pass on from master to disciple. Some plants are known to everyone, and if they are not indicated by an expert, it is not necessary to pay an initiation fee to know them. The position of herbalist can be purchased, and this is how one can be initiated to specialized secret knowledge about medicinal plants.”

It was also explained that because the herbalists’ activity brings them economic benefits, and because there are many women involved in this type of business, recipes and plants are jealously kept secret. In this study, many women interviewed attributed their success as herbalists to the fact that they knew plants and recipes unknown to others, which ensures good results in treatments of “difficult” diseases.

Usually, medicinal plant knowledge is passed down orally over generations through the females in the family, and strictly within the lineage, from mother to daughter during childhood and until puberty. Then, when the young woman marries, and is now considered to belong to her husband’s lineage [4, 67], it is her mother-in-law who continues to show her plants and recipes that she herself received from her own mother, if she has this ability. In fact, even if this type of knowledge is kept secret from outsiders, at each generation of women and marriage, knowledge transmission and enrichment continue, in a cumulative pattern.

The fact that most herbalists are women, coupled with the fact that the transmission pattern follows a female lineage might also explain the importance of plants used for women, and the higher use convergence noted for gynecological use of the same species. Finally, strict oral transmission within the lineage, apprenticeship through direct experience, and more importantly the economic value associated with this knowledge, which entails a need for its non-disclosure, as well as repeated household movement of families from one village to another [4] may be valid reasons to explain the non-sharing of plant names, as made evident in this study. Still, these results should be interpreted as preliminary. In future research, more herbalists, both women and men, should be interviewed, to gain a better understanding of this specialized knowledge transmission strategy, while putting this in perspective with the high economic value that medicinal plants now represent.

## Conclusion

The species collected in this study appear to be similar to those found in the dominant Lao lowland pharmacopeia previously described and to those already listed and well known in the Southeast Asian pharmacopeias [8–10, 12, 14, 15, 72]. We have highlighted the existence of a significant pool of pan-Asian or pan-tropical species with well-defined uses that have been already widely documented and even pharmacologically validated (i.e., *Centella asiatica*, *Bryophyllum pinnatum* (Lam.) Oken, *Senna alata*, *Aloe vera* (all used for various dermatological problems), *Psidium guajava* (diarrhea), *Embelia ribes* (anthelmintic), *Cynodon dactylon*, *Coix lacryma-jobi* (kidney problems), *Ganoderma lucidum* and *Anoectochilus lylei* (lung and liver problems, adjuvent cancer treatment), *Plantago major* L. (asthma), *Chromolaena odorata* (anti-hemorrhagic), *Eleutherine* spp., *Acorus calamus*, *Ageratum conyzoides*, *Eurycoma longifolia* Jack, *Blumea balsamiflora*, *Oroxylum indicum*, *Ficus hispida*, *Mucuna pruriens* (L.) DC., *Morus alba*, etc.). These data appear to contradict Anderson (1985) who noted that only a small number of the species collected in the remote Hmong mountain villages in Thailand are cited elsewhere but may be explained by Anderson’s prior publication of his work, and also by the place of study [28]. However, it would be interesting to continue these surveys in order to try to highlight whether there is a group of plants typically “Hmong,” emerging from the plants used by herbalists.

This study also points out that the Hmong use pattern of plants is very similar to that of traditional lowland Lao healers living mainly in southern Laos. In both groups, there is a predominant use of roots. In comparing preparation procedures of medicinal plants by the Hmong and other healers in Laos did not allow to tease out practices that can be interpreted as uniquely “Hmong.” Plants are prepared in form of decoctions, infusions, and used in steam baths and medicinal baths, or as culinary preparations with therapeutic properties (such as chicken broth, stuffed hearts or steamed stuffed omelets) that blur the line between food and remedy [73].

However, unlike lowland healers, Hmong do not use a technique of preparing remedies called *fon yaa*, which consists in rubbing the hard ingredients of the remedies (stem, bark, root, shell, horn, etc.) on a special stone in a back and forth movement, and mixing the particles deposited with some water that will be drunk by the patient. This technique is characteristic of southern Laos, and it is clearly linked to ancient Khmer influence (Vidal 1958), and is therefore not integrated into Hmong practices. Finally, another difference that has been noted in the use of this pharmacopeia concerns the way in which postpartum is treated, even if some postpartum plants might be similar to both groups.

Significant differences between the Hmong and lowland Lao traditional medicines can be seen in the aspects of gender, transmission methods, and renumeration. Lowland Lao medicine, strongly influenced by Theravada Buddhist tradition, is usually practised by men and transmitted through the male line; however, as for the Hmong, this may be variable. While Hmong medicine is passed down orally, lowland Lao medical knowledge may include a combination of manuscripts, written prescriptions, and oral transmission [11]. Unlike the Hmong, lowland Lao healers do not traditionally accept payment for treatments, believing it to prevent the effectiveness of the medicine, and so renumeration is at the discretion of the patients, as an “offering,” and medical consultations usually take place in their houses in a village, although there are also “medicine sellers.” Thus, the Hmong have a greater financial imperative to keep their knowledge secret, within their lineage. In contrast, for the Lao healers, there is likely to be greater sharing and homogeneity in knowledge [12], although the more esoteric practices are not revealed to others and specific recipes are usually kept secret.

Finally, it is important to underline the dynamic and evolving nature of the Hmong pharmacopeia and its uses. Indeed, besides the importance given to plants used for women and to male tonics that are very popular and which seem characteristic of Asian pharmacopeias, there is also a significant set of indications that are clearly in line with very contemporary health concerns: strokes, weight problems, metabolic disorders. This demonstrates that Hmong herbalists are committed to providing therapeutic solutions adapted to the needs of the local population. Hmong herbalists, therefore, appear as fully fledged actors of health in Lao society, and their knowledge and practice should be recognized and valued as such.

## Additional file

Additional file 1: Hmong medicinal plants uses. (DOCX 229 kb)
