# Supplementary material for: Hmong herbal medicine and herbalists in Lao PDR: pharmacopeia and knowledge transmission
Source: J Ethnobiol Ethnomed. 2019 Jun 13;15:27. doi: 10.1186/s13002-019-0307-2 (PMC6567612; doi:10.1186/s13002-019-0307-2)
Supplement: Supplementary file 1 — Hmong medicinal plants uses. (DOCX 229 kb) [file 13002_2019_307_MOESM1_ESM.docx]

| **Herbarium Voucher** | **Scientific name** | **Family** | **Hmong name** | **Part of plant used** | **Medicinal indication** | **Recipe** |
| --- | --- | --- | --- | --- | --- | --- |
| JMD 382 | *Acacia* sp. | Leguminosae | tshuaj nab tom | bark and leaf | Snake bite | Crush bark and leaves are applied as a poultice. |
| JMD 104 | *Achyranthes aspera* L. | Amaranthaceae | nroj kab ntsig | leaf | Scabby skin | Crushed leaves are applied on skin. |
| JMD 107 | *Acmella paniculata* (Wall. ex DC.) R.K.Jansen | Compositae | tshuam suab / ntshav khov | root, fruit | (1) To purify blood (2) Deshydration | (1) A root decoction is drank (3 cups/day). (Bad blood condition is dignosed by the healer by scratching the patient collarbone). (2) The fruit os eaten raw, or a decoction is prepared with the fruit and drank, against deshydration. |
| JMD 854 | *Acorus* sp. | Acoraceae | pawj ya | whole plant | Asthenia, weak legs or hands | Whole plant decoction used in steam bath over affected limb, also as a lotion. |
| JMD 014 | *Aerides* sp. | Orchidaceae | txiv do ntoo /  paj muv | whole plant | To enhance memory | A decoction of the whole plant is drunk. |
| JMD 4 | *Aeschynanthus lineatus* Craib | Gesneriaceae | qhib ntoos | leaf | (1) Broken bone or wound (2) skin sores | (1) Crushed leaves are heated in banana leaf and applied hot on affected part renewed until the pain is gone. (2) Leaves are boiled 30 min and decoction drank or used to rub or soak the sore area. |
| JMD 346 | *Ageratina adenophora* (Spreng.) R.M.King & H.Rob. | Compositae | nroj liab | root | Jaundice | Root decoction drunk all day long, with *Chromolaena odorata* (L.) R.M.King & H.Rob. |
| JMD 257 | *Ageratum conyzoides* (L.) L. | Compositae | tshuaj kab liab | root | Male sexual weakness | Roots are macerated in alcohol and drunk, or roots are cut in small pieces and eaten with meat. |
| JMD 3 | *Ageratum conyzoides* (L.) L. | Compositae | paj pum tshis | leaf | Wounds (haemostatic) | Crushed leaves are applied fresh on bleeding wound. |
| JMD 311 | *Ageratum conyzoides* (L.) L. | Compositae | pwm tshis liab | whole plant | Spasms and shivering of all body | The whole plant is crushed and macerated in cold water for a few minute, then the macerate is drunk. |
| JMD 009 | *Aglaonema simplex* (Blume) Blume | Araceae | nab ntsuab nce ntoo | root, stem and leaves | (1) Broken bone (2) Mastitis | (1) Root is crushed with a little alcool, then heated in banana leaf and aplied on wound. (2) Stem and leaves are crushed and wrapped in banana leaf. Poultice is applied warm onto the breast. |
| JMD 299 | *Aglaonema simplex* (Blume) Blume | Araceae | tshuaj plab hlauv | leaf | Swollen liver with icterus signs, loss of qppetite (for children) | Crush leaves, and apply them as a poultice on liver area. Renew every day. |
| JMD 337 | *Agrimonia pilosa* Ledeb. | Rosaceae | Neev dev | whole plant | Constipation | The whole plant is prepared in decoctionand drunk all day. |
| JMD 82 | *Allomorphia balansae* Cogn. | Melastomataceae | ntsuag thoov | root | Heart problem, weak heart | Root decoction is drunk. |
| JMD 298 | *Aloe vera* (L.) Burm.f. | Xanthorrhoeaceae | tshuaj kub nhyab | leaf | Burns | Peel the epidermis of one leaf and apply the jelly on burn. |
| JMD 714 | *Alpinia conchigera* Griff. | Zingiberaceae | qhaus ntsim txob / qhiav dev | rhizom | Ringworm | The rhizom os this edible plant is mashed and applied as a poulitice. Should not stay to long on affected part. |
| JMD 55 | *Alpinia galanga* (L.) Willd. | Zingiberaceae | qhaus ntsim txob | stem | Skin scratches | Slice of stem are rubbed on the scratches |
| JMD 290 | *Alpinia oxymitra* K. Sch. | Zingiberaceae | xyab txhim tsuas | rhizom | Low back pain, weakness, loss of apetite, anemia (in older people) | The tuberous root is eaten raw. |
| JMD 700 | *Alpinia* sp. | Zingiberaceae | vaj phai xiav plaw, | rhizom | Diarrhoea, stomach problems and intestinal cramps | Rhizom is finely grated or chopped,then infused for 5 min in a cup of boiled water and drunk. |
| JMD 343 | *Alstonia rostrata* C.E.C.Fisch. | Apocynaceae | ntoo kaus | root | Back pain | Root is cooked in broth with chicken and eaten. |
| JMD 77 | *Alstonia scholaris* (L.) R.Br. | Apocynaceae | zej kaus ntawv | leaf | Headache | Chopped leaves are applied on forehead and temples. |
| JMD 66 | *Amischotolype mollissima* (Bl.) Hassk. | Commelinaceae | rauws ntsuab | leaf | Infected wounds by maggots (for animals) | Three young leaves are crushed, and then tied around the neck of animals. This is said to prevent flies eggs to hatch. |
| JMD 56 | *Amomum* sp. | Zingiberaceae | qhaus plaub | root | Cough | Decoction to drink. |
| JMD 67 | *Amomum* sp. | Zingiberaceae | qhaus zoo txhaws qa | root | Hoarseness | Anis tasting roots are chewed, or a root decoction is drunk warm. |
| JMD 769 | *Ancistrocladus tectorius* (Lour.) Merr. | Ancistrocladaceae | tshuaj quav tawv | stem | Diarrhoea | A handful of stem pieces is placed in one liter of water and prepared in form of a decoction. |
| EE05 | *Angelica sinensis* (Oliv.) Diels | Apiaceae | mua jua | root | (1) Uterine infection (2) Haemmorhoids | Slice into small pieces, boil 5-10 mins and drink |
| JMD 869 | *Angiopteris ceracea* Aldrew | Marattiaceae | muas thij | root | Liver problem (cyst) | Chopped root heated in ashes are applied as poultice on affected area. |
| JMD 832 | *Anoectochilus lylei* Rolfe ex. Downie | Orchidaceae | mob siab mob ntsws / hawb na panrog / zig zawg | whole plant | (1) Hepatitis, liver or (2) lungs problems | (1-2) The whole plant is chopped and steamed with eggs and eaten |
| JMD 349 | *Aphaenandra uniflora* (Wall. ex G.Don) Bremek. | Rubiaceae | puj npaim | root | Diabetes | A piece of root is placed in water and boil for at least 30 minutes. The decocotion is drunk all day long. |
| JMD 35 | *Aphaenandra uniflora* (Wall. ex G.Don) Bremek. | Rubiaceae | puj npaim | leaf and stem | Tonic, for skinny people, to increase apetite. | Leaves and stems are added to meat broth, eaten. |
| JMD 727 | *Aphanamixis polystachia* (Wall.) R. Parker | Meliaceae | ntoo txiv plauv lauv | root or wood or fruit | (1) Appendicitis, (2) stomach acute pain, gastrtis | (1) The root or wood is prepared in deocotion and drunk; the very bitter fruit can also be eaten. (2) Gastritis and stomach problems: (1) *Hedyotis capitellata*  Wall. ex G.Don whole plant with root (720) and wood of *Senna alata* (L.) Roxb.(725) root and branch of *Embelia ribes* Burm. F (726) and wood of *Aphanamixis polystachia* (Wall.) R. Parker (727) are prepared in docotion, which is drunk |
| JMD 268 | *Apium graveolens* L. | Apiaceae | koj liab | leaf | Galactagogue | Leaves are cooked in broth with chicken and eaten. |
| JMD 730 | *Aporosa tetrapleura* Hance | Phyllanthaceae | txiv quav me es | root | (1) Snake bite (2) male sexual weakness | (1) Pieces of root er cut and rubbed on a stone with little water. The extract obtained is applieddirectly on the bite. (2) Roots of *Aporosa tetrapleura* Hance 730 and *Dillenia* sp. 731 and stems and root of *Solanum torvum* Sw. (748) are chopped and macer |
| JMD 863 | *Aporosa* sp. | Phyllanthaceae | ntoo teb npleg | root and stem | Constipation | Root and branch decoction drunk |
| JMD 124 | *Aquilaria crassna* Pierre ex Lec. | Thymelaeaceae | maj zoo | leaf | Stomach pain | Leaves are prepared in fomr of a decoction to drink. |
| JMD 728 | *Aralia* cf. *foliolosa* Seem ex. C.B. Clarke | Araliaceae | pos tauj tshiab | vine stem | Urinary problems | The stem is cut in pieces and a decoction is prepared with and drunk. |
| JMD 088 | *Archidendron clypearia* (Jack) I.C.Nielsen | Leguminosae | ntoo teb npleg / kua dis nplej soob | leaf | Toothache | Mouth baths with leaves decoction. |
| JMD 756 | *Ardisia crenata* Sims. | Myrsinaceae | txiv qaum liab | root | Male sexual weakness | A root alcoholic maceration is prepared and drunk |
| JMD 089 | *Ardisia* sp. | Myrsinaceae | tshuaj zoo kem plab | root | Slow and bloating digestion, dyspepsia | A decoction of the root is drunk. |
| JMD 802 | *Ardisia* sp. | Myrsinaceae | ko tsaug | whole plant | (1) General weakness, tonic for old as youth therapy, (2) Postpartum recovery | (1) Whole plant prepared in decoction or alcoholic maceration drunk. (2) Whole plant is cooked in chicken broth. |
| JMD 831 | *Ardisia* sp. | Myrsinaceae | tshuaj qab nplooj liab | leaf and root and stem | General weakness, male sexual weakness | *Derris* sp. (839) is mixed with *Ardisia* sp. (831) and prepared in alcoholic maceration or in decoction which is drunk |
| JMD 281 | *Asparagus filicinus* Buch.-Ham. ex D.Don | Asparagaceae | tshuaj quav miv | root | Kidney stone | The tuberous root is boiled until the water turns red. This preparation is drunk all day long for a few days. |
| JMD 098 | *Aspidopterys tomentosa* (Blume) A.Juss. | Malpighiaceae | hmab dawb | leaf | Damaged ligament, Sprain | Chopped leaves are applied on damaged tendon help healing. |
| JMD 75 | *Aspidopterys tomentosa* (Blume) A.Juss. | Malpighiaceae | txiv hmab ko tshob | leaf | Snake bite | Leaves are chopped are applied on the bite, or on the affected part. |
| JMD 873 | *Asplenium* sp. | Aspleniaceae | suab mob raj | leaf | Broken bone | Leaves are on softened ambers and applied on wound. This poultice is renewed 2 times a day until feeling better. |
| EE75 | *Basella alba* L. | Basellaceae | mab cav | aerial part | Haemorrhoids | Boil for 30 mins. |
| JMD 807 | *Bauhinia* sp. | Leguminosae | nplooj npaim liab | leaf and stem | Depurative, to increase urine flow, kidney stone | A decoction of leaves and stems is drunk |
| JMD 332 | *Bauhinia variegata* L. | Leguminosae | paj vuav tsav | bark | Diarrhea with blood in the stool (dysenteria) | Bark is prepered in decoction during 30 min and drunk all day. |
| JMD 743 | *Bidens* cf. *pilosa* L. | Compositae | nrhab koob | whole plant | Tooth decay | Root and stem of *Bidens cf. pilosa* L. (743) and *Blainvillea acmella* (L.) Philipson.(742) with whole *Scoparia dulcis* L. 744 plant are coocked in chicken broth. Preparation can be boiled 2 times. |
| JMD 745 | *Bischofia javanica* Blume | Euphorbiaceae | ntoo qaub | bark | Diarrhoea | Bark decoction drunk as drinking water all day. |
| JMD 742 | *Blainvillea acmella* (L.) Philipson. | Compositae | nrhab hob | whole plant | Tooth decay | Root and stem of *Bidens* cf. *pilosa* L.743 and *Blainvillea acmella* (L.) Philipson.(742) with whole *Scoparia dulcis* L. 744 plant are coocked in chicken broth. Preparation can be boiled 2 times. |
| JMD 718 | *Blumea balsamifera* (L.) DC | Compositae | zaj xaws / paj xiav / tov dev dawb | root | (1) Gonorrhoea, leucorrhoea, hemorrhoids (2) Contraceptive (used to space births) | (1) Root of Leea rubra (715), *Catunaregam tomentosa* (DC.) Tirveng. (716), *Blumea balsamifer*a (718), and an indetermined whole plant with root (717), are prepared in decoction, which is drunk.(2) The root of this plant is cooked with another root. |
| JMD 313 | *Boenninghausenia albiflora* (Hk.) Rchb. Ex Meissn. | Rutaceae | cag dawb | root | Loss of apetite | Root is prepared in form of a decoction to drink, or cooked in meat broth. |
| JMD 375 | *Boesenbergia longiflora* (Wall.) Kuntze | Zingiberaceae | qhaus | rhizom | Abdominal pain after sexual relation ( for men) | A whole rhizom is eaten raw. |
| JMD 705 | *Boesenbergia rotunda* (L.) Mansf. | Zingiberaceae | sab ntxim | rhizom | (1) Diarrhoea and painful stomach (2) Male sexual weakness, (3) Headache and muscular tension in the shoulder and neck (due to stress). | (1) Rhizoms are chopped in small pieces and eaten (2) Rhizoms are prepared in form of an alcholic maceration 3-Rhizom are mashed in form of a pate which is rubbed on the neck, occiput and temples. |
| JMD 331 | *Brassaiopsis ciliata* Dunn | Araliaceae | thoob huab | inner wood | Constipation | The soft part of the wood is infused in boiling water,for 10 min and drunk. |
| JMD 091 | *Bridelia stipularis* (L.) Blume | Euphorbiaceae | hmab lo qhov txhab | bark or leaf | Wounds or bruises or broken limb (traumas) | Chopped bark or crumpled leaves are applied on wounded areas to help healing, notably in the case of a broken bone. |
| JMD 57 | *Bridelia stipularis* (L.) Blume | Euphorbiaceae | hmab txua txhab | leaf | Broken bone | Leaves crumpled are applied around wounded area. |
| JMD 108 | *Broussonetia papyrifera* (L.) L'Hér. ex Vent. | Moraceae | cev pub npua | Root | Stomach pain, gastric ulcer | Root decoction is drunk. (Leaves are also given to pigs). |
| JMD 251 | *Bryophyllum pinnatum* (Lam.) Oken | Crassulaceae | kuab nplai taub | leaf | To prevent miscarriage | Leaves are chppoed with leaves of 250 (*Galium aparine* L.), stirred in beaten egg, steamed in banana leaf and eaten. |
| JMD 771 | *Buddleja asiatica* Lour. | Scrophulariaceae | tshau toj | leaf and terminal buds | Gastritis | Leaves and terminal buds are mixed with eggs or pork meat and steamed. |
| JMD 60 | *Byttneria aspera* Collebr. ex Wall. | Sterculiaceae | hmab txuas leeg | leaf | Sprain, tore ligaments | Leaves are crushed and applied onto the wound. |
| JMD 40 | *Calamus poilanei* Conrad | Arecaceae | kav theej npoom / nplooJ kav teej | sap | Mouth sores | The white sap which drips when stem is sliced is applied on mouth sores (this is a very efficient remedy) |
| JMD 819 | *Calocedrus macrolepis* Kurz | Cupressaceae | npeeb | wood | Genital herpes and other STI with painfull urination | The wood is chopped and the decoction is drunk. |
| JMD 716 | *Catunaregam tomentosa* (DC.) Tirveng. | Rubiaceae | pos kaus ntsaj | stem and root | Gonorrhoea, leucorrhoea, hemorrhoids | Root of *Leea rubra* (715), *Catunaregam tomentosa* (DC.) Tirveng. (716), *Blumea balsamifera* (718), and an indetermined whole plant with root (717), are prepared in decoction, which is drunk. |
| JMD 120 | *Cayratia japonica* (Thunb.) Gagnep. | Vitaceae | txiv tsuag taub | fruit or seed | (1) Swollen spleen (2) edible food | (1) Crushed fruits or seeds are applied on stomach area. (2) The leaves are boiled and eaten as a vegetable. |
| JMD 085 | *Celosia argentea* L. | Amaranthaceae | paj mab | root | Irregular menstruations | The root decoction is drunk. |
| JMD 334 | *Centella asiatica* (L.) Urb. | Apiaceae | lauj vag | whole plant | Dermatitis (ithcy skin with red spots), | Prepare a bath with the plant and rub te plant on itchy area. |
| JMD 70 | *Chassalia curviflora* var. *ophioxyloides* (Wall.) Deb & B.Krishna | Rubiaceae | yeeb qus | Root and wood | Painful urination (stones in the urinary tract) | Root and wood decoction is drank warm until pain is gone. |
| JMD 722 | *Cheilocostus speciosus* (J. Koenig) C.D. Specht | Costaceae | kos qeej | stem and rhizom | leucorrhoea with pelvic pain | Evodia sp.719- *Hedyotis capitellata* Wall. ex G.Don 720-*Urena lobata* L. 721 *Cheilocostus speciosus* (J. Koenig) C.D. 722 Specht -*Mussaenda* *pubescens* W.T. Aiton 723-*Clerodendrum schmidtii* C.B.Clarke 724. are prepared in decoction (until the color changes) |
| JMD 325 | *Chloranthus nervosus* Coll. & Hemsl. | Chloranthaceae | yas dub | root | High fever with shivering | Root is prepared in form of decoction durin 30 minutes and drunk all day. |
| JMD 296 | *Chlorophytum comosum* (Thunb.) Jacques | Asparagaceae | tshuaj fab | leaf | Allergic shock | Poor hot water on leaves and infuse 5 min then drink. |
| JMD 002 | *Chromolaena odorata* (L.) R.M.King & H.Rob. | Compositae | paj dawb | leaf | Wounds (haemostatic) | Crushed leaves with water are applied fresh on bleeding wound. |
| JMD 273 | *Chromolaena odorata* (L.) R.M.King & H.Rob. | Compositae | tshuaj paj dawb | Root | (1) Dysenteria (2) Cough | (1-2) Root are chewed with some water |
| JMD 370 | *Chrysopogon aciculatus* (Retz.) Trin. | Poaceae | nroj puav khab | whole plant | Kidney problem | Whole plant decoction is drunk all day. |
| JMD 32 | *Cinnamomum iners* Reinw. ex Blume | Lauraceae | ntoo xyab phij | bark | Shamanic use (incense) | Finely chopped bark is heated, gets sticky and rolled on small bamboo stick to use as incense |
| JMD 278 | *Cipadessa baccifera* (Roth) Miq. | Meliaceae | tshuaj cas cees | root | Gonorrhoea | The root is prepared in decoction (20 min) which is drunk lukewarm as drinking water. Treatment untill feeling better. . |
| JMD 277 | *Clausena excavata* Burm. f. | Rutaceae | tshuaj paj ntxoog | leaf | Cold with fever | Crush leaves applied on the internal part of the arm , on wrist and ankles. |
| JMD 365 | *Clausena excavata* Burm. f. | Rutaceae | txiv quav tshis | root | Post partum pain | A root decoction (30 min) is drunk all day long. In another recipe, the root is cooked in broth with chicken, as a meal. |
| JMD 78 | *Clausena lenis* Drake | Rutaceae | ntoo dev zi | leaf and root | Fever with shivering | Root is chopped and prepared in decoction, which is drunk. At the same time, leaves are chopped are applied on wrist and ankles. |
| JMD 327 | *Cleisostoma rostratum* Seidenf. ex Aver. | Orchidaceae | vaj txov ntoov | whole plant | Broken bone | *Cleisostoma rostratum* (Lindl.) Garay (327), and *Oberonia caulescens* Lindl. (328) are mashed together and applied in form of a poultice around broken bone area. |
| JMD 317 | *Clerodendrum glandulosum* Lindl. | Verbenaceae | ntshaub tsw | root | To avoid miscarriage | Cut 9 pieces of root (one finger length each representing one of the 9 parts of the body.Cchop, stir in eggs and steam in banana leaf. Eat two times. |
| JMD 5 | *Clerodendrum glandulosum* Lindl. | Verbenaceae | ntshaub rws ntsuab | leaf | (1) Skin rash (from contact with caterpillar), (2) Liver problem (tough liver) | (1) Leaves are smoked over the fire and rubbed on affected area. (2) Crushed leaves are applied cold onto the liver area. |
| JMD 724 | *Clerodendrum schmidtii* C.B.Clarke | Verbenaceae | zaub ntsuab ntshws qus / tshuaj ntsuab tsws | root and stem | leucorrhoea with low abdomonal pain | *Evodia* sp.719- *Hedyotis capitellata*  Wall. ex G.Don 720-*Urena lobata* L. 721 *Cheilocostus speciosus* (J. Koenig) C.D. 722 Specht -*Mussaenda pubescens* W.T. Aiton 723-*Clerodendrum schmidtii* C.B.Clarke 724. are prepared in decoction (until the color changes) |
| JMD 38 | *Cnestis palala* (Lour.) Merr. | Connaraceae | hmab mias | fruit | Stomach pain (light) | One fruit is eaten |
| JMD 368 | *Codonopsis javanica* (Blume) Hook.f. & Thomson | Campanulaceae | txiv qab hub | root | Male sexual weakness | The root is cooked in chicken broth and eaten. |
| EE57 | *Coix lacryma-jobi* L. | Poaceae | yeb caus | seed | Diuretic, to promote urination | Boil few seeds and drink. |
| JMD 781 | *Coix lacryma-jobi L.* | Poaceae | ntseb ntsaug | whole plant with seeds | Urinary problems with frequent miction, Kidney stone | Used alone in decoction, or a decoction is prepared with *Coix lacryma*-*jobi* L. (781) and indet (775) and drunk. |
| JMD 865 | *Colocasia esculenta* (L.) Schott | Araceae | tshuaj qos dub | stem | Accute abdominal pain | Two pieces of 3 cm of stem each are placed to boil in form of decoction which is drunk. In another recipe, stem is cooked in the ashes, masehd in a mortar aand aplied direclty on the abdomen. |
| JMD 280 | *Colocasia fallax* Schott | Araceae | tshuaj qab lwj hli | corm | Skin ulcer | The corm is crushed and applied in poultice on affected area, renewed during 2 or 3 days. |
| JMD 836 | *Comnbretum erythrophyllum* (Burch.) Sond. | Combretaceae | hmab nplawm qau | root | Male sexual weakness, general tonic | An alcoholic maceration of the root is drunk |
| JMD 701 | *Costus* sp. | Costaceae | ? Ne s'en souvient plus (ou peut etre meme nom que 700?) | rhizom | Diarrhoea, stomach problems and intestinal cramps | Rhizom is finely grated or chopped,then infused for 5 min in a cup of boiled water and drunk. |
| JMD 262 | *Crinum* sp. | Amaryllidaceae | tshuaj muaj me nyuam | bulb | Fertilty enhancer | Decoction of bulb is drunk. |
| JMD 379 | *Crinum* sp. | Amaryllidaceae | tw ntse dub | whole plant | Excessive chilliness | The whole plants prepared in decoction, or leaves are infused for 10 min. The preparation is drank all day. |
| JMD 265 | *Crotalaria assamica* Bth. | Leguminosae | vaj khaum nyeg | leaf | Stomach problem, dyspepsia | Leaves are cook with pork stomach or pork meat. |
| JMD 814 | *Croton* cf. *dongnaiensis* Pierre ex Gagnep. | Euphorbiaceae | luam laws zoov | leaf and root and stem | Eczema, skin swelling, allergic manifestations | Whole plant with root decoction is drunk |
| JMD 318 | *Cryptolepis dubia* (Burm.f.) M.R.Almeida | Asclepiadaceae | hmab kub tawn | leaf | Swollen and painful joints, sprain | Crushed leaves are used to make a poultice around the sore joint. |
| JMD 780 | *Curcuma comosa* Roxb. | Zingiberaceae | vaj khoos dawb | rhizom | (1) Gynecological infections (2) Hemorrhoids | (1) Gynecological infections: rhizom decoction drunk. (2) *Pandanus urophyllus* Hance (777) leaves and *Curcuma comosa* Roxb. (780) rhizom are prepared in decoction used in lotion or bath seat. |
| EE41 | *Curcuma* sp. | Zingiberaceae | qhia poj ntoov | rhizom | Intoxication (to prevent) | Take before eating or after eating (or) drinking |
| JMD 378 | *Curcuma* sp. | Zingiberaceae | qhaus ntsuab cog | rhizom | Stomach pain, gastritis (strong) | One rhizom is chopped and drunk with some water. |
| JMD 380 | *Curcuma* sp. | Zingiberaceae | qhaus nyeg | root | Constipation | Crush root are macerated for 5 min in cold water, which is drank 2 times a day. |
| JMD 752 | *Curcuma* sp. | Zingiberaceae | vas khoos daj | tuber | (1) Cervical and dorsal nevralgia (2) Female low abdominal pain | (1-2) Root is sliced and prepared in form of a decoction. (For sale, the root is dried and powdered) |
| EE20 | *Curcuma zanthorrhiza* Roxb. | Zingiberaceae | wan nang (lao) | rhizom | Uterine pain and infection | Take 3 slices of 'blue' and 'white' types, boil together for 15 mins. |
| JMD 702 | *Curcuma zanthorrhiza* Roxb. | Zingiberaceae | qhiav daj | root | Liver and gall bladder pain | Rhizom is gratted, prepared in decoction for 5', untill water turns yellow. To drink. Rhizom can be used dried and powdered. |
| JMD 28 | *Cuscuta* sp. | Convolvulaceae | nkauj rwm seb | whole plant | Asthenia, general weakness | Stems and leaves are boiled in water for inhalation (over 20 min under a towel). |
| JMD 879 | *Cyathula prostrata* (L.) Blume | Amaranthaceae | nrhab lo dev tw | aerial part | Urinary problem (difficulties to urinate and few urine), chronical intestinal pain, with spasms. | A decoction is made out of the plant (without the root) and drunk. |
| JMD 874 | *Cyclea peltata* (Lam.) Hook f. &Thomson | Euphorbiaceae | hmab cab liab | leaf and root and stem | (1) weak linb, paralysis (brain stroke consequences), (2) kidney stones | (1 )A decoction is prepared with the stem and leaves of indet. (853), stem of (indet.) 870, *Cyclea peltata* (Lam.) Hook f. &Thomson (874) whole plant , root and branch of indet. (879) bis and is drunk. (2)Boil the whole plant (roots, stem and leaves) and |
| JMD 735 | *Cynodon dactylon*  (L.) Pers. | Poaceae | nyom | whole plant | Urinary problems (frequent mictions) | A deocoction is prepared with Lophaterum gracile (737), *Polygonatum* sp. (739), *Cynodon dactylon* (735) an undetermined plant (712) and insect nest building material and drunk. |
| EE10 | *Dalbergia bariensis* Pierre | Leguminosae | pa dong daeng (lao) | bark | Rheumatism, pain in the joint | Boil 2 inches of bark in 1 litre of water for 15 mins, drink as needed |
| JMD 010 | *Dalbergia oliveri* Prain | Leguminosae | tshuaj hluas Nplooj | root and young branches | Cough with blood | A decoction is made with the root and the young branches and drunk lukewarm. |
| JMD 884 | *Debregeasia longifolia* (Burm.f.) Wedd. | Urticaceae | nplias | root and stem | To loose weight | Root and stem of *Debregeasia* *longifolia* (Burm.f.) Wedd. (884) are mixed with *Nepenthes* sp. (824) roots and prepared in a decoction which is drunk. |
| JMD 110 | *Decaneuropsis cumingiana* (Benth.) H.Rob. & Skvarla | Compositae | kas las mas paj daj | whole plant | (1) Sore legs, (2) Itchy eyes | (1) Legs are bathed with a plant decoction. (2) A decoction made out of the whole plant is is used as a lotion for the eyes. |
| JMD 114 | *Decaneuropsis cumingiana* (Benth.) H.Rob. & Skvarla | Compositae | hmab daj | aerial part | Itchiness, infected rash | Leaves and stems are crushed and squeezed. The juice extracted is rubbed onto the affected skin. |
| JMD 377 | *Deeringia amaranthoides* (Lam.) Merr. | Amaranthaceae | tshuaj zoo nhyuv tws | root | Strong intestinal pain, said to be due to food poisoning | The root is prepared in decoction (20 min) which is drunk all day long. |
| JMD 58 | *Derris* aff. a*cuminata* Benth. | Leguminosae | hmab cuav pos hmab pos | wood, bark | (1) Stomach pain (ulcer), (2) broken bone | (1) Chopped wood decoction is drunk daily. (2) Bark is chopped and wrapped around broken bone area. |
| JMD 839 | Derris sp. | Leguminosae | hmab tsuaj pob | vine stem | Male sexual impotency, general weakness | Derris sp. (839) is mixed with Ardisia sp. (831) and prepared in alcoholic maceration or in decoction which is drunk |
| JMD 708 | *Desmodium gangeticum* (L.) DC. | Leguminosae | tshuaj ka / ntsua av | whole plant | (1) Cough (2) urinary problem (frequent mictions) | (1-2) The whole plant is prepared in decoction, until color of the water changes and is drunk. |
| JMD 340 | *Desmodium multiflorum* DC. | Leguminosae | tshuaj qhov muag pom kev | root | Bad eyesight | Root decoction drunk all day. |
| JMD 710 | *Desmodium* sp. | Leguminosae | tshuaj txiaj npib | root and stem | (1) Leucorrhoea, (2) Loss of appetite, (3) male sexual weakness, | (1-2) Root and stem are prepared in decoction until the color of the water changes and is drunk (3) Root in form of an alcoholic maceration |
| EE37 | *Desmodium triquetrum* (L.) DC. | Leguminosae | tsua ntxoj | leaf and stem | Heart problems |  |
| JMD 295 | *Dianella ensifolia* (L.) DC. | Xanthorrhoeaceae | tshuaj pob kws | Root | Stomach pain, inflamation of the stomach, after eating spoiled food. | The root is cooked in broth with chicken and eaten. |
| JMD 65 | *Dianella ensifolia* (L.) DC. | Xanthorrhoeaceae | aub laug ntxuam | bark and root | To enhance digestion and stomach potency | Decoction of root and bark is drunk. |
| JMD 113 | *Dichrocephala integrifolia* (L.f.) Kuntze | Compositae | co kem | Root | Opium addiction | A root decoction of this plant is drunk daily. |
| JMD 731 | *Dillenia* sp. | Dilleniaceae | doesn't know hmong name | root | Male sexual weakness | Roots of *Aporosa tetrapleura* Hance 730 and *Dillenia* sp. 731 and stems and root of *Solanum torvum* Sw. (748) are chopped and macerated 2 days in alcohol and drunk (3 small liquor glass 3 times a day, around one week) |
| EE70 | *Dioscorea cirrhosa* Lour. | Dioscoreaceae | Bao leuak (lao) | tuber | Blood in stools | Boil the tuber for 20-30 mins. |
| JMD 825 | *Dioscorea hispida* Dennst. | Dioscoreaceae | sab yaj thawj | tuber | Severe diarrhoea | The tubers are chopped and prepared in form of a decoction which is drunk. This decoction ca also be adminstered in form of enema for children or vbabaie sunbale to drink . The tuber can also be eaten raw.  Young tuber have a lumpy appearance, the old on |
| JMD 842 | *Dioscorea* sp. | Dioscoreaceae | hmab cab qaib | tuber | Limb paralysis | The chopped tuber is chopped and boiled. The resulting decocotion is used to lotion the affected part . |
| JMD 086 | *Diploclisia glaucescens* (Bue.) Diels | Menispermaceae | hmab txiaj | leaf | (1) Dermatitis (without itchiness), (2) Sprain | (1) Leaves crumpled are rubbed onto the skin. (2) leaves are crushed and wrapped around the wounded joint. |
| JMD 297 | *Dipteracanthus repens* (L.) Hassk. | Acanthaceae | kuas txob tshuab | leaf | High fever with cold feeling | Hot water is poured on leaves, and let to infuse until water turns red. To drink. |
| JMD 254 | *Disporum calcaratum*  D. Don | Colchicaceae |  | ND | Cough | Boil with other plants and drink 1 glass 2-3 times a day. |
| JMD 805 | *Dracaena surculosa* Lindl. | Asparagaceae | nplooj xyoob ntsuab | leaf and root and stem | Low back ache with general asthenia | A deocotion is drunk. |
| JMD 872 | *Drynaria quercifolia* (L.) J. Sm. | Polypodiaceae | suab laun ntoo | root | Fibrotic liver, kidney deficiency | A deocoction is prepared with the chopped root and drunk until cured (said to be a very good plant for liver and kidney). |
| JMD 253 | *Eclipta prostrata* (L.) L. | Compositae | paj daj | leaf | Heart weakness | A pig heart is stuffed with *Eclipta prostrata* (L.) L. 253 and *Gynura* sp. 252 and steam the lot and eat. Or the plants are beaten with egg, which is steamed in banana leaf and eaten. |
| JMD 713 | *Elephanthopus scaber* L. | Compositae | tshuaj kuab plog | root | Cough | A decoction is prepared with the root and drunk |
| JMD 887 | *Eleutherine* sp. | Iridaceae | tshuaj txoob ntug liab | bulb | Bleeding stomach ulcer, male sexual weakness | The bulb is prepared in alcoholic maceration or decoction drunk check pour impuissance masculine/ulcère de l'estomac (sang dans l'estomac) check si aussi |
| JMD 707 bis | *Eleutherine subaphylla* Gagnep. | Iridaceae | txoob ntug liab / ຫົວຜັກບົ່ວເລືອດ | bulb | (1) Stomach ulcer, swelling of lynph nodes (mumps, groin), (2) Postpartum recovery | (1) Stomach ulcer: the bulb decoction is drunk, or the bulb can be steamed with eggs and eaten. (2) The bulb is cooked in chicken broth is eaten. |
| JMD 354 | *Elsholtzia winitiana* Craib | Lamiaceae | maj qus | leaf | Otitis with pus, ear ache | Fewh leaves are crushed the juice is squeezed in the water. |
| JMD 50 | *Embelia parviflora* Wall. ex A.DC. | Myrsinaceae | hmab peb cauj nplooj | root | Diarrhoea | Root of this liana are prepared in from of a deccotion which is drunk in the case of liquid diarrhea. |
| JMD 726 | *Embelia ribes* Burm. f | Primulaceae | hmab ntsuag xyas | root and stem | (1) Gastritis (2) Taenia worm | (1-2) *Embelia ribes* Burm f. (726), *Hedyotis capitellata*  Wall. ex G.Don 720- *Senna alata* (L.) Roxb.725, *Aphanamixis polystachia* (Wall.) R. Parker 727, are chopped and prepared in form of a decoction which is drunk. |
| JMD 34 | *Embelia sessiliflora* Kurz | Myrsinaceae | hmab ntsuag sas | stem | Abdominal pain | A decoction of the ligneous stem of this liana is drank. |
| JMD 351 | *Emilia sonchifolia* (L.) DC. ex DC. | Compositae | tshuaj paj daj | whole plant | Abces | The whole plant is crushed and beaten in eggs, the steamed in banana leaf and eaten |
| JMD 818 | *Eriocaulon buergerianum* Bunch.-Ham.ex Mart. | Eriocaulaceae | paj nav nras | aerial part | Bad eye sight | Flower and stem are prepared in form of a decootion which is drunk |
| JMD 778 | *Eriocaulon sexangulare* L. | Eriocaulaceae | paj nras | aerial part | Bad eye sight | A decoction of the whole plant is drunk all day. This treatment is repeated until feeling better. |
| JMD 45 | *Eryngium foetidum* L. | Apiaceae | nplooj hmiav kaw / puam hub tuaj pos | whole plant | Venous problem, blood clots. | A decoction made ot of the whole plant is drunk warm. |
| JMD 025 | *Erythrina subumbrans* (Hassk.) Merr. | Leguminosae | pos nrhav | bark | Broken bone | A long piece of bark is cut out and rolled around the wounded area. Or the bark is crushed and applied on. |
| JMD 770 | *Erythropalum scandens* Blume | Menispermaceae | hmab qab los | root | Loss of apetite | Root are chopped and prepared in form of a deocotion, which is drunk. |
| JMD 741 | *Euphorbia pulcherrima* (Willd. Ex Klotzsch) Graham | Euphorbiaceae | tshuaj paj qos ntoos | Flower bud | Galactagogue | Few flowers buds, picked up before blossoming are boiled in chicken broth. |
| JMD 315 | *Eurya acumminata* DC. | Theaceae | ntoo maj soob | leaf and root and stem | Allergic dermatitis resulting from the contact of aspecific tree sap. | Root, bark, wood or leaves ((together or separatly) are prepared in decoction during 30 min, then used for bath or to lotion affected area. 2-3 aplplications are enough to cure problem. |
| JMD 817 | *Eurycoma longifolia* Jack | Simaroubaceae | ntoo zes qaib | bark or root or wood | Joint stiffness and pain | Root (better) or wood, or bark are chopped and prepared in dcocotion. Some people also make pills with this plants. |
| EE09 | *Eurycoma longifolia* Jack. | Simaroubaceae | hak yen dong (lao) | root | malaria | Combine with *Tinospora crispa* stem for malaria (10 cm of each, sliced). Boil together, drink as necessary. (Can also be manufactured as tonic) |
| JMD 363 | *Eurysolen gracilis* Prain | Lamiaceae | tws zis | whole plant | Mental confusion and asthenia | The whole plant is prepared in docotion, which is drunk all day long. |
| JMD 37 | *Ficus hirta* Vahl | Moraceae | cev | sap, root | (1) wounds. (2) Urinary problem | (1) The white sap which drips when leaves are pulled out is applied on open wounds. (2) Root decoction is drunk |
| JMD 729 | *Ficus hirta* Vahl | Moraceae | ntoo cev | root | Loss of appetite | The root is cooked in chicken broth. |
| JMD 43 | *Ficus hispida* L. f. | Moraceae | cev xyoob | root | Nerve damage, hemiplegia, paralysis | Root decoction is drunk and used also for baths. |
| JMD 845 | *Ficus* sp. | Moraceae | tshuaj vaj khaum | stem and root | Weak limbs, joint stifness | *Psychotria samentosa* Blume (8O1), *Ficus* sp. (845) stem and roots and indet. (815) are used in steam bath applied over affected limbs |
| JMD 864 | Ficus sp. | Moraceae | hmab vaj khaum | vine stem | Rheumatic pain, stiff joints | A stem decoction is drunk. |
| JMD 353 | *Firmiana simplex* (L.) W.Wight | Malvaceae | txiv thoog yug loj | Leaf and fruit | To protect from white ghosts or spirits coming to tease and bother people (specially when you leave near a cemetary) | Put leaves under your bed pillow or above your bed. The fruit is also hanged around the house. |
| JMD 49 | *Flemingia macrophylla* (Willd.) Merr. | Leguminosae | cev soob | whole plant | Urinay problem (interrupted urine flow) | Whole palnt wit roots is prepared in decoction to drunk |
| JMD 250 | *Galium aparine* L. | Rubiaceae | kuab nplai dib | leaf | Miscarriage prevention | Leaves are chppoed with leaves of 251 (Kalanchoe pinnata (Lmk.) Pers.), stirred in beaten egg, steamed in banana leaf and eaten. |
| JMD 835 | *Ganoderma lucidum* (Curtis) P. Karsten | Polyporaceae | nceb liab | whole fungus | Liiver problem, lungs problem, diabetes | The whole fungus is prepared in decoction and drunk. |
| JMD 69 | *Garcinia merguensis* Wight | Clusiaceae | tshuaj muaj zog | bark or wood | (1) Tiredness like after long walk, (2) male sexual impotency | (1-2) Bark or wood is sliced and placed in maceration in alcool, to drink |
| JMD 012 | *Glochidion rubrum* Blume | Euphorbiaceae | maj mum soob | root, leaf | (1) Cough with blood. (2) Bleeding gums, teeth and gum decay, mouth sores, bad breath | (1) Root decoction drank luckwarm. (2) Leaves are crumpled and rubbed onto the gums / mouth sores or chewed . |
| JMD 087 | *Glochidion rubrum* Blume | Euphorbiaceae | nruj xem | leaf | Mouth sores | Leaves are chewed are applied locally. |
| JMD 866 | *Glochidion* sp. | Phyllanthaceae | tshuaj liab ntsis | leaf | Burns | Leaves are chopped, quickly dried over a flame and applied as a poultice on burn. |
| JMD 859 | *Gnetum* sp. | leguminosae | hmab peb dis | leaf | Broken bone | Chopped leaves are applied as a poultice on wound, which is renewed regularly every 2 hours (up to one month). |
| JMD 7 | *Gomphostemma wallichii* Prain | Lamiaceae | cag rog | root | Stomach ache with blood in feces or diarrhoea | Decoction of root is drunk luckwarm |
| JMD 324 | *Gomphrena celosioides* Mart. | Amaranthaceae | tshuaj keej ntuj | whole plant | Tireness in old person, after hard work, general weakness with low back pain | The whole plant is cooked in broth with meat or is macerated in alcohol. |
| JMD 47 | *Goniothalamus macrophyllus* (Blume) Hook.f. & Thomson | Annonaceae | ntoo txhab ntshav | leaf and stem | Loss of appetite | Leaves and branches aree placed in the kettle for daily drinking water. |
| JMD 856 | *Gouania javanica* Miq. | Rhammaceae | hmab saw nyaiaj | leaf and root and stem | Limb paralysis, weakness (possibly due to brain stroke consequences). | (1) A decoction is prepared Gouiana javanica (856) whole plant, Cyclea peltata (Lam.) Hook f. &Thomson (874) whole plant , and three undetermined plants (853, 870, 879bis) and is drunk. |
| JMD 084 | *Grangea maderaspatana* (L.) Poir. | Compositae | cos kev | aerial part | Fever | Plant is chopped and rubbed on the skin behind the knee and in the elbow folding |
| JMD 747 | *Grewia retusifolia* Kurz | Malvaceae | ntoo nplias cf. note | stem (bark and wood) | Gastritis | Chopped wood and bark are prepared in decoction and drunk. |
| JMD 750 | *Gynura japonica* (Thunb.) Juel | Compositae | nroj rog / nroj nyo haus | leaf and stem | Burns | Leaves and stems are are wrapped in a banana leaf and heated in ambers, then banana leaf is opened and preparation applied as a warm poultice. |
| JMD 252 | *Gynura* sp. | Compositae | tshuaj ob yam nplooj | whole plant | Heart weakness | A pig heart is stuffed with *Eclipta prostrata* (L.) L. and *Gynura* sp. (252) and steam the lot and eat. Or the plants are beaten with egg, which is steamed in banana leaf and eaten. |
| JMD 720 | *Hedyotis capitellata* Wall. ex G.Don | Rubiaceae | kuab taw nyuv ntsuab / hmab txog siav | whole plant | (1) Gastritis, stomach problem (2) Leucorrhoea with pelvic pain | (1) *Hedyotis capitellata* Wall. ex G.Don whole plant with root (720) and wood of *Senna alata* (L.) Roxb.(725) root and branch of *Embelia ribes* Burm. F (726) and wood of *Aphanamixis polystachia* (Wall.) R. Parker (727) are prepared in docotion, which is drunk |
| JMD 29 | *Hedyotis capitellata* var. *pubescens* Kurz | Rubiaceae | kuab taws hnyuv xiav | aerial part | Sore leg (muscle or stiffness when awakening). | The leaves of this poisonous plant are crushed and applied fresh on sore leg |
| JMD 81 | *Hedyotis capitellata* var. *pubescens* Kurz | Rubiaceae | ntub yag | root | Fever with shivering | Chopped root is applied on wrist and ankles |
| JMD 877 | *Hedyotis* sp. | Rubiaceae | tshuaj zoo taub hau txias | whole plant | Persistent headache without fever | whole plant decoction drunk. |
| JMD 099 | *Helicteres hirsuta* Lour. | Sterculiaceae | nroj tob puam | root | Abdominal pain, strong stomach pain without burning feeling | The root is eaten raw or boiled and the decoction is drunk. |
| EE06 | *Helicteres isora* L . | Sterculiaceae | tau jue | fruit and stem | Intestinal infection, dysentery | Slice into small pieces, fry or boil, or make a powder |
| JMD 800 | *Heterotis* sp. | Melastomataceae | tshuaj qaub | whole plant | Gall bladder stone | A whole plant decoction is drunk |
| JMD 274 | *Holcoglossum subulifolium* (Rchb.f.) Christenson | Orchidaceae | tshuaj zoo ceg txham | whole plant | Stiff legs, walking difficulties | The whole plant is boiled to be used to lotion the lesg and also as a drink (1 liquor glass morning and evening). |
| JMD 761 | *Homalomena occulta* (Lour.) Schott | Araceae | teeb nyug | root | Limb numnbness, paralysis of old people, brain stroke consecuency | The root is prepared in dcoction with other plants and is drunk |
| JMD 381 | *Homalomena* sp. | Araceae | nplooj taum faj | Leaf or root | Skin ulcer | Leave or root are crushed and applied in form of a poultice on affected area. This poultice is kept for one day and changed once. |
| JMD 001 | *Illigera trifoliata* (Griff.) Dunn ssp. cucullata (Merr.) Kub. | Hernandiaceae | hmab qis tes | leaf | Sore joints | Freshly picked up leaves are crushed and wrapped in a banana leaf and heated over the fire. This poultice is applied hot on sore or bruise joints. |
| JMD 42 | *Imperata cylindrica* (L.) Raeusch. | Poaceae | nqeeb | root | Sore throat | Root decoction is drunk. |
| JMD 751 | *Iresine herbstii* Hook. | Amaranthaceae | nca liab | whole plant | (1) Amenorrhea (2) Leucorrhoea with pelvic pain | (1) Whole plant decoction drunk as drinking water for 3 days (2) Decoction with *Mussaenda* sp. (766), *Iresine herbstii* Hook. (782) with 3 undetermined plants (760, 762, 764) is prepared and drunk. |
| JMD 255 | *Iris collettii* Hk. f. | Iridaceae | oob noog ntxuam dawb | ND | (1) Stomach problems, gastritis (2) to gain weight in very skinny people | Cook in broth with chicken. |
| JMD 51 | *Itea macrophylla* Wall. | Iteaceae | nplooj zuaj mis | leaf | Mastitis during breast feeding | Leaves are gently heated above fire and the breast is massaged with. |
| JMD 093 | *Jasminum nervosum* Lour. | Oleaceae | paj zuam nyuj | leaf | Ear problems | Leaves are crushed and squeezed above ears to let few drops of liquid drip in them. |
| JMD 267 | *Justicia ventricosa* Wall. | Acanthaceae | tshuaj tu leeg | ND | Low blood pressure and varicose veins | A decoction is drunk |
| EE54 | *Kaempferia cochinensis* Gagn. | Zingiberaceae | tsawb ntwg | rhizom | Bloating after eating | Eat small pieces fresh. |
| EE47 | *Kaempferia galanga* L. | Zingiberaceae | puas toj | rhizom | Headache from thinking too much, healing after operation | Prepare a wash with the rhizom and eat a small piece. |
| JMD 706 | *Kaempferia galanga* L. | Zingiberaceae | pua toj nyeg | rhizom | Stomach and intestine bloating | Rhizom decoction of *Kaempferia* sp (704), aznd *Kaempferia galanga* (706) is drunk. |
| EE04 | *Kaempferia parviflora* Wall. Ex Baker | Zingiberaceae | xab tchim du | rhizom | Stomach ache | Chew the fresh rhizome (also manufactured into tablets : for lao people it is a tonic) |
| JMD 707 | *Kaempferia parviflora* Wall. Ex Baker | Zingiberaceae | qhiav dub | rhizom | (1-2) Diarrhoea, stomach ulcer (3) male sexual weakness | (1-2) Rhizom is eaten raw. (3) Rhizoms of *Boesenbergia rotunda* (L.) Mansf. (705) are mixed with *Kaempferia parviflora* (707) and prepared in alcoholic maceration. A small glass is drunk. |
| JMD 269 | *Kaempferia rotunda* L. | Zingiberaceae | xab txhim suav | root | Limb stiffness, paralysis, general weakness | Boil the root to lotion the body. |
| JMD 376 | *Kaempferia* sp. | Zingiberaceae | qhau liab cog | root | Stomach pain (especially in case of alcohol drinking) | The root macerated in alcohol, and drunk. |
| JMD 704 | *Kaempferia* sp. | Zingiberaceae | tsawb ntug ntsuab / xam yaum ntsuab | rhizom | Stomach and intestine bloating | Rhizom decoction of *Kaempferia* sp. (704) and *Kaempferia galanga* (706) drunk. |
| JMD 827 | *Kalanchoe* sp. | Crassulaceae | tshuaj nplaum | whole plant | Wounds | Whole plants mashed and used in poultice on wound which is renewed twice a day. |
| JMD 68 | *Lasianthus attenuatus* Jack | Rubiaceae | nees nkaum tus nyuj / sob tshuaj zoo mob laug | root and stem | Tonic for old people | Root and stem are added to chicken broth taken during 1 or 2 days. |
| JMD 41 | *Leea indica* (Burm. f.) Merr. | Vitaceae | nplav ntxaiv | stem, leaf | (1) Painful urination (2) Wound haemostatic | (1) A decoction of pieces of stem is drank warm. (1-2 cups a day for 3 days). (2) Chopped leaves are applied on wound. |
| JMD 715 | *Leea rubra* Blume ex Spreng. | Vitaceae | plav ntxaiv / paj iab qaib nras | root | Gonorrhoea, leucorrhoea, hemorrhoids | Only big roots are used for this plant. Root of *Leea rubra* (715), *Catunaregam tomentosa* (DC.) Tirveng. (716), *Blumea balsamifera* (718), and an indetermined whole plant with root (717), are prepared in decoction, which is drunk. |
| JMD 826 | *Lepisanthes rubiginosa* (Roxb.) Leenh. | Sapindaceae | tshuaj quav me es | root | (1) Flatulence (2) men sexual weakness | (1) Root decoction drunk. (2) Root alcooholic maceration is drunk. |
| EE21 | *Ligusticum striatum* DC. | Apiaceae | tshab xyoob | root | (1) Stomach ache, poor appetite (2) Uterine pain | Boil piece of root for 30 minutes and drink. |
| JMD 352 | *Limnocharis flava* (L.) Bucheneau | Alismataceae | paj khuam teeb | bulb | Low blood pressure (orthostatic presssure) | A entire bulb are prepared in decoction for 30 min, which is drunk all day long. |
| JMD 364 | *Litsea* *cubeba* (Lour.) Pers. | Lauraceae | zaw kos | sap from wood | Dermatitis (red spots) with fever | Pieces of wood are placed on ambers he sap dripping out is collected in a glas and drunk. |
| JMD 837 | *Litsea cubeba* (Lour.) Pers. | Lauraceae | ntoo txiv kos | stem | Children cough and fever | A decoction of a piece of stem is drunk |
| JMD 737 | *Lophatherum gracile* Brongn. | Poaceae | zab zis | root | Urinary problems | A deocoction is prepared with *Lophaterum gracile* (737), *Polygonatum* sp. (739), *Cynodon dactylon* (735) an undetermined plant (712) and insect nest building material and drunk. |
| JMD 738 | *Ludwigia* sp. | Onagraceae | siv txham | whole plant | Infected wound | The whole plant chopped and applied in poultice for 2 days, which is renewed 1 times a day. |
| EE39 | *Lycopodium cernuum* L. | Lycopodiaceae | cag suab | aerial part | Back pain | Boil in a small amound of water (1 glass) and drink |
| JMD 31 | *Lycopodium cernuum* L. | Lycopodiaceae | suab tshws | aerial part | Yellow skin, anemia, general waekness | Leaves and stems are boiled in water for inhalation (the patient breathes the steam under a blanket for over 20 min). |
| JMD 851 | *Lycopodium squarrosum* G.Forst. | Lycopodiaceae | fwj txwj zaj | aerial part | Bloating, swollen stomach | Stem and leaves are steamed with eggs and eaten |
| JMD 732 | *Lygodium conforme* C. Chr | Polypodiaceae | cag suab dub / suab xov tooj | root | Nevralgia | A decoction of the root is drunk. |
| JMD 292 | *Lysionotus serratus* D.Don | Gesneriaceae | tsw zis | root | Male sexual weakness | The root is crushed root mixed with chopped meat, or boiled in the water and eaten. |
| JMD 886 | *Macaranga* sp. | Euphorbiaceae | ntoo qiaj ntsuab | stem | Rheumatic pain, stiff joints | A decoction of a pice of stem is prepared and all day long drunk. The stem can be reused 3 times. |
| JMD 020 | *Maesa permollis* Kurz | Primulaceae | kab yeeb tshuaj | leaf | (1) Burns, (2) deshydration resulting from diarrhea | (1) Leaves crushed are applied fresh on burns. (2) Leaves are boiled and decoction drank. |
| JMD 275 | *Mahonia napaulensis* DC. | Berberidaceae | tshuaj ntoo nkaub qe | bark | Wounds, haemostatic | The bark of stem s scrapped and applied on wound. |
| JMD 860 | *Mallotus barbatus* Müll.Arg. | Euphorbiaceae | huab ncuas tuaj plaub | root | Ovarian cyst | *Mallotus barbatus* Müll. Arg (860), *Wendlandia* sp.( 861) and indet. (862) are prepared in docction and drunk until cured. |
| EE15 | *Medinilla septentrionalis* (W. W. Smith) H. L. Li | Melastomaceae | hmab tsua sa | leaf | Fever (hot) | Boil (1 bag makes 2 litres). |
| JMD 111 | *Melastoma malabathricum* L. | Melastomataceae | ncaim toj liab | root | (1) Contraceptive (2) Edible | (1) A root decoctionis drank by women during their periods to prevent getting pregnant. (2) Fruits are black berries which are particularly appreciated by birds, squirrels and also children who eat them when passing by. |
| JMD 360 | *Melastoma malabathricum* L. | Melastomataceae | toj ncaim | root | Diarrhoea | A decoction of *Psidium guajva* L. is prepared with *Melastoma malabathricum* L. ssp. *normale* (360) roots and drunk daily. |
| JMD 369 | *Melicope pteleifolia* (Champ. Ex Benth.) T.G. Hartley | Rutaceae | ntoo kaws dawb | bark and wood and root | Post partum pain | Bark, wood, root are prepared in form of a decoction for 20 min, which is drunk all day long. |
| JMD 719 | *Melicope pteleifolia* (Champ. Ex Benth.) T.G. Hartley | Rutaceae | zaub ntshis kaws / zaub twj kum | stem | Leucorrhoea with pelvic pain | *Evodia* sp.719- *Hedyotis capitellata* Wall. ex G.Don 720-*Urena lobata* L. 721 *Cheilocostus speciosus* (J. Koenig) C.D. 722 Specht -*Mussaenda* *pubescens* W.T. Aiton 723-*Clerodendrum schmidtii* C.B.Clarke 724. are prepared in decoction (until the color changes) |
| JMD 345 | *Microglossa pyrifolia* (Lam.) Kuntze | Compositae | nroj paj dawb | root | Gonorrhea | Root decoction used to lotion male sexual organ or woman sexual organs |
| JMD 847 | *Microglossa pyrifolia* (Lam.) Kuntze | Compositae | tshuaj paj daj | leaf and root and stem | Genital infection with leucorrhea, mucous membrane irritation | The whole plant with root is prepared in decoction and drunk |
| JMD 62 | *Millettia caerulea* Baker | Leguminosae | txiv quav me es | root | (1) To enhance stomach potency (2) Baby rash | (1) Root decoction is drunk warm. (2) Root decotion is applied as a lotion |
| JMD 011 | *Millettia extensa* (Benth.) Baker | Leguminosae | hmab tsw nyuj | sap, leaf and stem | (1) Sore or wounded eye, (2) hemiplegia or paralysis | (1) Slice the stem and allow the sap to drip into the eye. The effect occurs within 30 min. (2) Leaves and stem are prepared in decoction drunig 1 hour and warm baths (1 hour) are administered twice a day for over a month. |
| JMD 52 | *Millettia extensa* (Benth.) Baker | Leguminosae | hmab tsw nyuj | sap | Wounded eye | Vinestem is sliced above the eye to let the sap drip into it. |
| JMD 64 | *Millettia pachycarpa* Benth. | Leguminosae | ntoo txiv taum nyiaj | root | Hemorrhagic diarrhoea | Root decoction is drunk warm, 2-3 times a day until diarrhea stops. |
| JMD 820 | *Millettia* sp. | Leguminosae | hmab ntshas ou ntshav | stem | Post partum : placenta delivery | A piece of stem is cooked in chicken broth and drunk. |
| JMD 338 | *Mimosa pudica* L. | Leguminosae | tshuaj tsaug zog | whole plant | Limb numbness with local oedema | The whole plant is prepared in decoction and drunk all day. |
| JMD 746 | *Mimosa pudica* L. | Leguminosae | pos tsaug zog | whole plant | Insomnias, urinary problem (frequent urination) | (1); The plant is mixed wit other plant and prepared in form of a decoction (2) A decoction is prepared and drunk. |
| JMD 73 | *Molineria latifolia* (Dryand. ex W.T.Aiton) Herb. ex Kurz | Hypoxidaceae | nplooj qhaib xyab | root | Painful urination (stones in the urinary tract) | Root decoction is drunk. |
| JMD 336 | *Momordica cochinchinensis* (Lour.) Spreng. | Cucurbitaceae | txiv tsuag taub | seed | Strong abdominal pain, spasms | The seed is chopped finely, and half of it is eaten raw. At the same time, One full seed is grinded on a stone with some water. The resulting juice is applied on the abdomen. |
| JMD 018 | *Morinda angustifolia* Roxb. | Rubiaceae | yeeb qus | leaf, root | (1) Painful urination, (2) local body sore | (1) For painful urination root decoction, drank hot. (2) Quickly heat the leave above fire, then rub into the skin. |
| JMD 829 | *Morinda* sp. | Rubiaceae | tshuaj ntoo daj | root | Low back ache | A decoction made out of the root is drunk |
| JMD 333 | *Morus alba* L. | Moraceae | zaub kab | Leaf or stem | Stomach ache and weakness after eating spoiled food | Leaves are prepared in form of an infusion, or stems or small branche sin form of decoction, which is drunk. |
| JMD 840 | *Mucuna pruriens* (L.) DC | Leguminosae | txiv taum txaij | seed | Snake bite | The seed is parted in twp, then rubbed on a stone with little water and the juice is applied on bite. |
| JMD 125 | *Musa* sp. | Musaceae | tsawb txho / tsawb quaw miv | root | Diarrhea with vomiting (enteritis), food poisoning | The collar jonction between roots and stem is crushed and the liquid extracted drank. |
| JMD 723 | *Mussaenda pubescens* Dryand | Rubiaceae | ntoo npauj npaim dawb | stem | leucorrhoea with pelvic pain | *Evodia* sp.719- *Hedyotis capitellata* Wall. ex G.Don 720-*Urena lobata* L. 721 *Cheilocostus speciosus* (J. Koenig) C.D. 722 Specht -*Mussaenda* *pubescens* W.T. Aiton 723-*Clerodendrum schmidti*i C.B.Clarke 724. are prepared in decoction (until the color changes) |
| JMD 838 | *Mussaenda sanderiana* Ridl. | Rubiaceae | hmab npauj npaim dawb | stem | Leucorrhoea with pelvic pain | A decoction of a piece of stem is drunk, until feeling better |
| JMD 767 | *Mussaenda* sp. | Rubiaceae | hmab tsw quav | stem | Blocked stomach (feeling full without eating, painfull) | A decoction of a piece of stem is drunk |
| JMD 092 | *Mycetia gracilis* Craib | Rubiaceae | zoo mob txeeb zig | Root | Painful urination (stones in the urinary tract) | Root decoction is drunk all day long. |
| JMD 824 | *Nepenthes* sp. | Droseraceae | tshuaj paum twm | root | To loose abdominal fat | Root and stem of *Debregeasia longifolia* (Burm.f.) Wedd. (884) are mixed with *Nepenthes* sp. (824) roots and prepared in a decoction which is drunk. |
| JMD 357 | *Nephrolepis undulata* (Afzel.) J. Sm. | Nephrolepidaceae | suab roob zeb | tuber | Skin ulcer | Tubers are crushed and applied in form of a poultice on ulcer. |
| JMD 328 | *Oberonia caulescens* Lindl. | Orchidaceae | oob noog ntxuam | whole plant | Broken bone | *Cleisostoma rostratum* (Lindl.) Garay (327), and *Oberonia caulescens* Lindl. (328) are mashed together and applied in form of a poultice around broken bone area. |
| JMD 362 | *Oldenlandia pinifolia* (Wall. ex G.Don) Kuntze | Rubiaceae | tshuaj ka | whole plant | Toothache | Add to beaten egg. Then steam in banana leaf and eat. |
| JMD 804 | *Ophiopogon japonicus* (L.f.) Ker Gawl. | Asparagaceae | pawj zoov | whole plant | Post partum complications | The whole plant decoction is drunk |
| JMD 821 | *Ormosia laosensis* Niyomdham | Leguminosae | txiv taum niaj | leaf and stem | Leucorrhea with itchiness | Leaves and stems decoction is used as genital shower |
| JMD 326 | *Ornithochilus difformis* (Wall. ex Lindl.) Schltr. | Orchidaceae | tshuaj txuas leeg | whole plant | To help restore the circulation of blood after a trauma, or a broken limb. | Whole plant is crushed and used in poultice around affected place. |
| JMD 341 | *Oroxylum indicum* (L.) Bth. ex Kurz | Bignoniaceae | tshuaj zoo txiav | Bark and leaf | Swollen and painful liver with difficult breathing, no jaundice (liver cirrhosis, liver fibrosis) | Bark decoction drunk all day long. In addition, leaves are chopped in beaten eggs and steamed in banana leaf to be eaten. |
| JMD 876 | *Oxalis corniculata* L. | Oxalidaceae | ntsuas ntsev | whole plant | Whooping cough | An infusion is prepared with the whole plant (like a tea)which is drunk untill feeling better. Or the leaves are cokked and eaten. |
| JMD 121 | *Pandanus* sp. | Pandanaceae | puv luj qus / xub yib | fruit | Stomach pain | Fruit are dried then crushed and boiled. Resulting water is drunk. |
| JMD 777 | *Pandanus urophyllus* Hance | Pandanaceae | puv luj qus / xub yib qus | fruit, root | (1) Urinary problem (frequent urination), (2) placenta delivery (3)haemorroids | (1) Decoction with *Pandanus urophyllus* Hance fruit (777) and root of Smilax sp. 763 and indet. (757) root is drunk.(2) Placenta delivery : decoction of P*andanus urophyllus* Hance root (777) with and root of Smilax sp. (763) and leaves of indet. (757) (3) *Pandanus urophyllus* Hance (777) leaves and *Curcuma comosa* Roxb. (780) rhizom are prepared in decoction used in lotion or bath seat. |
| JMD 844 | *Pandanus urophyllus* Hance | Pandanaceae | nplooj txiv puv luj qus | leaf | (1) Hemorrhoids, (2) to ease childbirth, (3) against hypertension | (1-2-3) Leaves are prepared in decoction and drunk |
| JMD 276 | *Paraboea* sp. | Gesneriaceae | dawb hau / npauj nyiag | leaf and root | Gatsritis, stomach ulcer | Laves are chopped to stuff a pig stomach with, which is cooked and eaten. Also a decocotion is prepared with the root , to drink. |
| JMD 291 | *Peperomia pellucida* (L.) Kunth. | Piperaceae | tshuaj pob tsuas | whole plant | Foot swollen with red skin | The whole plant is crushed applied as poultice on affected part. |
| JMD 264 | *Peristrophe lanceolaria* (Roxb.) Nees | Acanthaceae | nca liab | aerial part | Gynecological problem : metrorrhagias | Boil in water and drink. |
| JMD 875 | *Persicaria barbata* (L.) H. Harav | Polygonaceae | txiv quav yeeb qus | leaf and root and stem | Post-partum complication | Whole plant with root is cooked in chicken broth is eaten. |
| JMD 095 | *Persicaria chinensis* (L.) H. Gross | Polygonaceae | ntsuag qaub dev | root | Stomach pain with nausea | A decoction, made out of the roots, is drunk. |
| JMD 372 | *Persicaria nepalensis* (Meisn.) Miyabe | Polygonaceae | tshuaj zoo mob ntswj | leaf | Lungs problems, infected lung and difficult breathing | A leaf decoction is drunk all day, or the plant is steamed with beaten eggs and eaten. |
| JMD 320 | *Peucedanum siamicum* Craib | Apiaceae | tshuaj rog | root | For skinny and weak people,to get weight and strength | The root is chopped, mixed with sliced meat and the lot is steamed in banana leaf. |
| JMD 259 | *Phaulopsis dorsiflora* (Retz.) Santapau | Acanthaceae | tshuaj txiv neej | aerial part | male sexual weakness | It is said to be a very good plant for this type of problem. Cook in broth with chicken or pork. |
| JMD 019 | *Phlogacanthus curviflorus* (Wall.) Nees | Acanthaceae | txiv qws pam | leaf | Headache, light fever | Leaves are crushed and and applied fresh on the inside of the wrists or ankles. The poultice is kept for one day. |
| JMD 306 | *Phrynium pubinerve* Bl. | Maranthaceae | nplooj ntse nyeg | root | Headache, hangover, diziness, desorientation, due to Intoxication by contact with toxic chemicals | A piece of the raw root is eaten. |
| JMD 59 | *Phrynium pubinerve* Bl. | Marantaceae | nplooj ntse liab | root | Headache, hangover, diziness, desorientation, due to Intoxication by contact with toxic chemicals | Root decoction is drunk. |
| JMD 6 | *Phrynium pubinerve* Bl. | Marantaceae | nplooj ntse liab | whole plant (with fruit) | Headache, hangover, diziness, desorientation, due to Intoxication by contact with toxic chemicals | Decoction of root and fruit drank cool (one big root for one liter). |
| JMD 868 | *Phrynium pubinerve* Bl. | Marantaceae | nplooj ntse ntsuab | root | Genital infection, gonorrhea | Root decoction of *Rubus alceaefolius* Poir. (867) and *Phrynium pubinerve* Bl. (868) is drunk until the person is cured |
| JMD 709 | *Phyllanthus emblica* L. | Euphorbiaceae | txiv kua dis nplej / txiv khawb | wood, fruit | Diabetes | A decoction of the wood and fruit is drunk. |
| JMD 711 | *Phyllanthus* sp. | Phyllanthaceae | ntoo kab ziam | stem | Constipation | Piece of stem of *Phyllanthus* sp. (711) are mixed with *Piper* sp. (849) to prepare a decoction which is drunk. |
| EE02 | *Picria fel-terrae* Lour. | Linderniaceae | La yu | aerial part | Malaria | Make a powder, drink 3 x per day /Boil or soak in water for 1 hour and drink (Lao people use as energy tonic, when macerated in alccol) |
| JMD 103 | *Piper sarmentosum* Roxb. | Piperaceae | lwj yem qus | leaf | (1) To keep good teeth (2) preventive (for animals) | (1) It Is chewed regularly to keep good teeth. (2) When pigs eat it, it is said to protect them against diseases. |
| JMD 366 | *Piper* sp. | Piperaceae | hmab fwj txob | whole plant | Gastritis, stomach ulcer | The whole plant is prepared in decoction (20 min) and drunk all day long. |
| JMD 849 | *Piper* sp. | Piperaceae | hmab plaub | whole plant | (1) Blood circulation (2) Constipation, intestinal pain | (1) A decoction is prepared withe the whole plant and drunk (2) Branch and wood of *Phyllanthus* sp. (711) are mixed with *Piper* sp. (849) to prepare a decoction which is drunk. |
| JMD 310 | *Plantago major* L. | Plantaginaceae | zaub ntswg npua | whole plant | Difficult breathing with purring | The whole plant is prepared in decoction, drunk 3 times a day. |
| JMD 808 | *Pogonatherum paliceum* (Lam.) Hack | Poaceae | nyom teeg | whole plant | Post-partum complication | A decoction made out of the whole plant plant is drunk. |
| EE03 | *Polyalthia suberosa* (Roxb.) Thwaites | Annonaceae | chuaj muaj kua mi | root | Galactagogue | Boiled, 1 handful 1 litre |
| JMD 739 | *Polygonatum* sp. | Asparagaceae | kuab plog | root | Urinary problems | A deocoction is prepared with *Lophaterum gracile* (737), *Polygonatum* sp. (739), *Cynodon dactylon* (735) an undetermined plant (712) and insect nest building material and drunk. |
| JMD 53 | *Pothos scandens* L. | Araceae | ntshuas nce ntoo | leaf and stem | Kidney problems, kideny stones | Leave and stem decoction is drunk all day for one week. |
| JMD 881 | *Pothos scandens* L. | Araceae | hmab ntshuas nce ntoo | whole plant | (1) Productive cough with blood phlegm lung problem (2) Limb weakness, paralysis, which might result from brain stroke consequencies | (1) Whole plant decoction drunk (2) Whole plant used in steam bath. |
| JMD 123 | *Premna coriacea* var. *villosa* (C.B.Clarke) A.Rajendran & P.Daniel | Verbenaceae | los hws kis hmab | root | Dizziness | Root decoction is drunk. |
| JMD 094 | *Pseuderanthemum latifolium* B. Hansen | Acanthaceae | ncaim toj liab | root | Contraception | In order to have a contracpetive effect, root decoction is drunk during menstruations. |
| JMD 100 | *Psidium guajava* L. | Myrtaceae | txiv cuab thoj | Leaf or root | Diarrhoea | Young leaves are eaten or a decoction prepared with the root is drunk. |
| JMD 361 | *Psidium guajava* L. | Myrtaceae | cuab thoj | root | Diarrhoea | A decoction of *Psidium guajva* L. is prepared with *Melastoma* *malabathricum* L. ssp. *normale* (360) roots and drunk daily. |
| JMD 801 | *Psychotria samentosa*  Blume | Rubiaceae | kuab caws | leaf and root and stem | Weak limbs, joint stiffness | *Psychotria samentosa* Blume (8O1), *Ficus* sp. (845) stem and roots and indet. (815) are used in steam bath applied over affected limbs |
| JMD 090 | *Psychotria* sp. | Rubiaceae | tshuaj phwv sab | root | Diarrhoea | Root decoction drank all day long. (This medicine is said to come from Lao people, hence the name of the plant). |
| JMD 71 | *Pteridium aquilinum* (L.) Kuhn | Dennstaedtiaceae | suab maum dev | leaf bud | Epistaxis | Young fiddleheads of this fern are placed into nostril. |
| JMD 105 | *Pueraria alopecuroides* Craib | Leguminosae | hmab soo | vine stem | To avoid spirits coming in one's house | When one fears spirits , 3 knots are made on this vine stem and put it onthe bed as a protection. |
| EE08 | *Reynoutria japonica* Houtt. | Polygonaceae | qaub nraug | root | fever (not malaria), rash | Slice and boil 5-10mins, to drink |
| JMD 80 | *Rhamnus napalensis* (Wall.) Laws. | Rhamnaceae | txiv ntsia ruas soob | root | Snake or other bites (insects etc.) | Chopped leaves are applied on bite, and root decoction is drunk |
| JMD 330 | *Rohdea tonkinensis* (Baill.) N.Tanaka | Asparagaceae | tshuaj pob kws | root | Stomach ache (empty stomach) | Root is chopped and chewed on with a little of water. |
| JMD 347 | *Rosa cymoa* Trat. | Rosaceae | paj ntshuas nplai | root | Low back pain with colored urine | A root decoction is drunk all day. |
| EE11 | *Rourea minor* (Gaertn.) Alston | Connaraceae | Mab qau (hmong) ya nang deng (lao) | vine stem | Detoxification, Stomach ache, pain, after eating | Slice the liana stem and boil |
| JMD 867 | *Rubus alceifolius* Poir. | Rosaceae | pos txiv qaum liab | root | Genital infection, gonorrhea | Root decoction of *Rubus alceaefolius* Poir. (867) and indet. (868) is drunk until te person is cured |
| JMD 102 | *Rubus blepharoneurus* Cardot | Rosaceae | pos tauv | whole plant | Weakness, anemia | The whole plant put in boiling water for inhalation, under a blanket. |
| EE38 | *Saccharum officinarum* L. | Poaceae | kab cib ntsav | stem | Dysenteria, blood in stool | Take for 2 days- The first day pices of stem are eaten raw. The second day they are 2nd boiled and drunk |
| JMD 115 | *Salvia misella* Kunth | Lamiaceae | nroj ta baw | leaf and stem | High fever | This plant tastes very bitter, so stems and leaves are just deeped a few seconds in boiling water which is then drank. |
| JMD 026 | *Sambuccus javanica* Blume | Adoxaceae | mos hav | leaf | (1) Traumatism with swelling of the body (2) Abdominal pain (tough abdomen) | (1-2) Leaves are crushed and applied fresh on swollen area |
| JMD 600 | *Sambuccus javanica* Blume | Adoxaceae | mos hav | leaf | (1) sprains, broken bones, (2) placenta delivery and post partum | (1) Crushed leaves of *Sambuccus javanica* (600) and *Vernonia* sp.(703) and applied as a poultice on affected area.  (2) To ease placenta delivery: leaves of *Sambuccus javanica* (600) and *Vernonia* sp.(703) are chopped and cooked in chicken broth. |
| EE45 | *Sapindus mukorossi* Gaernt. | Sapindaceae | txiv pau los | fruit | Goiter | Goiter (or tumor on throat). Rub on outside |
| EE31 | *Schefflera elliptica* (Blume) Harms | Araliaceae | Leb mue nang | leaf petiol | (1) Nerve damage in hands/ numbness in hands (2) To remove fishbones stuck in throat, hiccups | (1-2) Boil 2 pieces, drink as needed (combine with ya na noi and kheua an on) |
| JMD 850 | *Schefflera elliptica* (Blume) Harms | Araliaceae | tes poj ntxoog | petiol | Fishbone stuck in throat | The hand shapped petiol is separated from the branch and used to scrap the throat with. Or the petion is placed in water to boil. The resulting liquid is drunk. In order to get rid out of fishbone. |
| JMD 878 | *Scleria* sp. | Poaceae | tauj npaig / noog tsua los | leaf | Skin (cosmetic use, beautiful skin) | The yellow part of the leaves are prepared in decoction, which is used as a lotion and drunk. |
| JMD 282 | *Scleria terrestris* (L.) Fassett. | Cyperaceae | ncaug | root | Fever and skin outbreak in samll children | The root decoction is used as lotion. |
| JMD 74 | *Scoparia dulcis* L. | Scrophulariaceae | nplooj qab zib | leaf | (1) Mouth and tongue sores, (aphta), (2) Headache with fever. | (1) Leaves are chewed (2) Fruits are boiled and the decoction is drunk. |
| JMD 744 | *Scoparia dulcis* L. | Scrophulariaceae | nroj qub teb | whole plant | (1) Itchy skin outbreak all over the body with fever (2) Tooth decay | (1) *Scoparia dulcis* L. (744) whole plant, *Trema orientalis* (L.) Blume (734), and *Morus alba* L. leaves are boiled and drunk. (2) Root and stem of *Bidens* cf. *pilosa* L. (743) and *Blainvillea acmella* (L.) Philipson.(742) with whole *Scoparia dulcis* L. (744 |
| JMD 358 | *Scurrula ferruginea* (Jack) Danser | Loranthaceae | tshuaj zoo txia | whole plant | Painful swollen joints in skinny people | Leaves, wood and bark decoction to drink and use to lotion joints area. |
| JMD 774 | *Scurrula ferruginea* (Jack) Danser | Loranthaceae | ntoo tsob | whole plant | Productive cough with breath shortness | One handful of whole plant is placed one liter water and boiled.The decoction is drunk. |
| JMD 109 | *Selaginella helferi* Warb. | Selaginellaceae | suab liab | leaf | Dermatitis (itchy skin) | Dried leaves are finely chopped and cooked in pork fat with some gunpowder. Preparation is tied in a tissue to patch itchy areas. |
| JMD 322 | *Selaginella inaequalifolia* Spring. | Selaginellaceae | suab tshws | whole plant | Swollen limb, oedema | The whole plant is prepared in decoction (20 min ) then used to lotion or bath affected area. |
| JMD 736 | *Selaginella* sp. | Selaginellaceae | suab | whole plant | (1) Varicoses veins (2) Nevralgia | (1-2) A steam bath is prepared In association with other plants. |
| JMD 279 | *Selliguea* sp. | Drynariaceae | suab pob tsuas | root | Swollen and painful liver | Root are crushed and applied in poultice, changed every day during 3 days. |
| JMD 846 | *Semecarpus anacardium* L. f. | Anacardiaceae | txhais liab | inner wood | Ovarian cyst | Remove the bark. Chop red inner part of wood and use in form of a steam bath. |
| JMD 725 | *Senna alata* (L.) Roxb. | Leguminosae | Hmong only know it's lao name | leaf, wood | (1) Ringworm (2) Gastritis and stomach problems | (1) Leaves are crushed and applied in poultice on the affected part. (2) Gastritis and stomach problems: (1) *Hedyotis capitellata* Wall. ex G.Don whole plant with root (720) and wood of *Senna alata* (L.) Roxb.(725) root and branch of *Embelia ribes* Burm. F (726) and wood of *Aphanamixis polystachia* (Wall.) R. Parker (727) are prepared in docotion, which is drunk |
| JMD 339 | *Senna hirsuta* (L.) H.S.Irwin & Barneby | Leguminosae | tshuaj kau tawv | root | Local oedema | Root is placed to boil in water for 30 min. This decocotion is drunk all day. |
| JMD 303 | *Sisyrinchium palmifolium* L. | Iridaceae | zab yi liab | root | Internal haemorrhage, trauma | Pieces of roots are prepared in broth with chicken and eaten. |
| EE13 | *Smilax glabra* Roxb . | Smilacaceae | ya hua (lao) | Tuber | (1) Birth recovery, post partum (2) Tonic | (1-2) Slice the tuber and boil for 5-10mins. To drink (tonic after chlid birth) |
| JMD 013 | *Smilax ovalifolia* Roxb. Ex. D. Don | Smilacaceae | hmab tshuaj txuas leeg | stem, leaf | (1) Wounded ligament or tendon traumas, or (2) large and profound wounds | (1) Stem or young tip is crushed, heated in banana leaf and applied cool locally (2) Crushed leaves and wrapp them fresh around the wound. Change this poultice every 2 days, for 2 weeks. This plant is used to put back together the skin in case of a cut on |
| JMD 342 | *Smilax ovalifolia* Roxb. Ex. D. Don | Smilacaceae | tshuaj txiv neej muaj zog | root | Male sexual weakness | Root is soaked in alcohol. One small glass is drunk daily. |
| JMD 367 | *Smilax* sp. | Smilacaceae | qos sab yaj tawj | tuber | Dysenteria | The tuber, when big is chopped and prepared in decoction for 20', which is drunk all day long. (The tuber when cooked is also edible). |
| JMD 763 | *Smilax* sp. | Smilacaceae | sab yaj thauj hmab | root, stem | (1) Stomach bloating, (2) urinary poblems (frequent urnation) (3) placenta delivery | (1) Stems are prepared in decoction and drunk until feeeling better . (2) Decoction with *Pandanus urophyllus* Hance fruit (777) and root of *Smilax* sp. 763 and indet. (757) root is drunk. (3) |
| JMD 307 | *Solanum* sp. | Solanaceae | vaj khaum nyeg | leaf | (1) Fever, (2) Male sexual weakness | (1) Fever : Poor hot water over the leaves and drink. (2) A pig stomach is stuffed with this plant and eaten. |
| EE16 | *Solanum spirale* Roxb. | Solanaceae | kuab tsib dai | bark | Hypertension | Boil slices of bark and drink. |
| JMD 284 | *Solanum spirale* Roxb. | Solanaceae | tshuaj kub nhyab | root, leaf | (1) Kidney stone, difficulties to urine and swollen bladder (2) Burns | (1) A root decoction drank (said to be very efficient ) (2) Leave are crushed and the juice is squeezed on burns. |
| JMD 304 | *Solanum spirale* Roxb. | Solanaceae | kuab tsib dai | leaf and root and stem | For a cold, with runny nose | The whole plant is prepared in decoction for 10 min and drank. |
| JMD 748 | *Solanum torvum* Sw. | Solanaceae | pos quav nkais | stem, root | (1) Diabetes, (2) Male sexual weakness | (1) A stem decoction is drunk. (2) Roots of Aporosa tetrapleura Hance 730 and Dillenia sp. 731 and stems and root of Solanum torvum Sw. (748) are chopped and macerated 2 days in alcohol and drunk (3 small liquor glass 3 times a day, around one week) |
| JMD 017 | *Solanum verbascifolium* L. | Solanaceae | sa ku cha / ntoo tshuaj pom | fruit and/or leaf, root | (1)To get rid of leeches (for buffaloes) (2) sore throat when swallowing, (3) sharp pain in lower abdomen | (1) When buffalos stay too long in water, they may get leeches in their nose and cough. Fruits and /or leaves of this treelet are crushed and squeezed, and the liquid extracted aplied in nostrils. (2-3) A root decoction is made and drank (or the root is cthe root is crushed and applied on throat. |
| JMD 096 | *Spermacoce remota* Lam. | Rubiaceae | zoo kho mob hniav | root | Toothache | The is root chopped finely, cooked in little water with beef is eaten. |
| JMD 30 | *Spermacoce remota* Lam. | Rubiaceae | kuab taws hnyuv dawb | leaf | Sore leg (muscle or stiff when wake up). | Leaves are crushed and applied fresh on sore leg |
| JMD 371 | *Sphagneticola trilobata* (L.) Pruski | Compositae | tshuaj zoo plawv | whole plant | For heart problems, heart weakness | Steam the whole plant with chicken liver in banana leaf and eat. |
| JMD 289 | *Stephania* sp. | Menispermaceae | hmab ntsha | root | Male sexual weakness | crush root and apply poultice on abdomen (zoo cem phev , txiv neej) |
| JMD 118 | *Sterculia lanceolata* Cav. | Sterculiaceae | seb | root or seed | Stomach and oesofagus ulcer | Seeds are baked in ashes and eaten, or a root decoction is prepared to drink. |
| JMD 46 | *Sterculia lanceolata* Cav. | Sterculiaceae | txiv kaus seb | root | Constipation | Root decoction to drink. |
| JMD 883 | *Sterculia lanceolata* Cav. | Sterculiaceae | ntoo seb | stem | (1) Male sexual weakness (2) high blood pressure | (1) The stem of *Ziziphus attopoensis* Pierre (857) is mixed with *Sterculia* *lanceolata*Cav. (858) and prepared in form of a decoction which is drunk (2) A decoction made out of the stem is drunk |
| JMD 286 | *Streblus* sp. | Moraceae | tshuaj maknao hav zoov | Root and wood | Pelvic pain with abundant leucorrhea, gonorrhea | Root and wood are prepared in decoction (30 min), drunk 3 times a day during 1 week. |
| JMD 772 | *Streptocaulon juventas* (Lour.) Merr. | Apocynaceae | hmab tshuaj qos ntoos | root | Stomach trouble after drinking or eating | The gratted root is eaten raw. |
| JMD 823 | *Strychnos angustifolia* Benth | Loganiaceae | hmab txeeb zig | root and stem | Kidney problem : turbid urine with sand, Kidney stones | Root and stem are prepared in decoction and drunk |
| JMD 882 | *Syzygium* sp. | Myrtaceae | txiv hle ceg | stem | To loose weight | A decoction is prepared with a 10 cm piece of stem placed in 1l water drunk. (The stem can be boiled 3 times). |
| JMD 61 | *Syzygium tetragonum* (Wight) Wall. ex Walp. | Myrtaceae | txiv cuab thoj qus | bark or root bark | Cough with blood | A bark decoction (20 min) is drunk and used to rub the chest. |
| JMD 63 | *Tabernaemontana bovina* Lour. | Apocynaceae | ntoo kaus taw qaib | leaf and root and stem | (1) Gonorrhoea, (2) Toothache | (1) Whole plant decoction is drunk 3 times a day, during 2 weeks. (2) Peels of root are applied on the tooth in order to desinfect and relieves the pain affected areas. |
| JMD 83 | *Tabernaemontana bovina* Lour. | Apocynaceae | kaus taw qaib | bark, leaf and stem | (1) Skin disease (2) Itchiness on all or part of body, allergic reaction. | (1) Bark is cooked in porc fat, and rubbed on affected areas.(2) Leaves and stem decoction is used to bath or wash the affected part. |
| JMD 765 | *Tacca* sp. | Taccaceae | nplooj qhwv yeeb | root | (1) High blood pressure (2) Diabetes | (1-2) One handful of underground part is sliced, dried boiled and drunk |
| JMD 300 | *Talinum fruticosum* (L.) Juss. | Talinaceae | kuab nplai taub | leaf | Dystocic childbirth | When there is contractions, but the baby wont come out. Poor hot water on leaves, allow to infuse and drink lukewarm. |
| JMD 021 | *Tetrastigma laoticum* Gagnep. | Vitaceae | tshuaj tsib nploog | leaf | Bruises | Leaves crushed are applied fresh on bruises. |
| JMD 48 | *Tetrastigma* sp. (T. pyriforme Gagnep. ?) | Vitaceae | hmab xwm leej ntsuab | Root | Cold, with sneezing and runny nose. | The root decoction is drunk. (Fruit is edible) |
| EE07 | *Thalictrum dioicum* L. | Ranunculaceae | lia tsua | root | (1) Diabetes and liver disease, (2) eye disease | (1) Boil and drink. (2) Boil and put water directly into eye. |
| JMD 830 | *Thottea tomentosa* (Blume) Ding Hou | Aristolochiaceae | tshuaj hli | whole plant | gall bladder inflamation, hepatitis | A decoction made out of the whole plant is drunk |
| JMD 246 | *Thunbergia grandiflora* (Roxb. ex Rottl.) Roxb. | Acanthaceae | paj haus kua | root and stem | Abortive, to recover after delivery (post partum) | The decoction of chopped root and stems is drank by women to abort or after giving birth to help to recover quickly |
| JMD 126 | *Thysanolaena latifolia* (Roxb. ex Horn.) Honda | Poaceae | tsuav mob miv, tsaub txho, tauj khaub rhuab (grass broome) | root | Galactagogue | Root decoction drank 2/3 tlmes a day. |
| EE33 | *Tinospora crispa* (L.) Hook. f. & Thomson | Menispermaceae | kheua khao hoy (lao) | vine stem | Malaria, diabetes | Slice and boil 5-10 mins, combine with *Eurycoma longifolia* for malaria- Take 10cm of each, slice and boil together for 5-10mins, drink as necessary until feel better |
| JMD 740 | *Tithonia diversifolia* (Hemsl.) A. Gray | Compositae | paj iab | leaf bud and stem | (1) Fever in chidren (2) Stomach problem | (1-2-) Leafbud and stem are crushed, packed in banana leaf. The lot is heated on ambers and applied as poultice on the abdomen. It is renewed 2 times a day. |
| JMD 272 | *Toddalia asiatica* (L.) Lam. | Rutaceae | tshuaj tsw nquas | root | Traumas with bruises or internal haemorrhage | The root decoction is drank. |
| JMD 344 | *Toddalia asiatica* (L.) Lam. | Rutaceae | txiv quav ntswg | root | Prostatic problem | In case of serious problem : a root decoction is drunk all day. |
| JMD 72 | *Toddalia asiatica* (L.) Lam. | Rutaceae | pos tsib npuas | root | Constipation | Root decoction is drunk |
| JMD 350 | *Torenia violacea* (Azaola. ex Blanco) Penn. | Scrophulariaceae | nroj paj xiav | whole plant | Constipation | Whole plant decoction drunk all day. |
| JMD 312 | *Toxicodendron rhetsoides* (W. G. Craib) Tardieu | Anacardiaceae | yuj | bark or root or wood | Infection in children : fever with red spots and difficult breath | Part of root, or bark or wood are prepared in decocotion to drink all day. |
| JMD 734 | *Trema orientalis* (L.) Blume | Cannabaceae | ntoo maj | inner wood | Itchy skin outbreak all over the body with fever (mostly on children) | *Scoparia dulcis* L. (744) whole plant, *Trema orientalis* (L.) Blume (734), and *Morus alba* L. leaves are boiled and drunk. |
| JMD 027 | Trevesia palmata (Roxb. ex Lindl.) Vis. | Araliaceae | thoob huab | leaf | Mastitis during breast feeding | Leaves crushed are applied hot on the breast. |
| JMD 261 | *Typhonium trilobatum* (L.) Schott | Araceae | xiab pwm chom | root | (1) High blood pressure (2) Hoarseness | (1) Leaves are chopped and cooked with an egg and eaten (2) The root is crushed, stirred into water and drunk. |
| JMD 112 | *Uncaria macrophylla* Wall. | Rubiaceae | pos kub yaj ntsuab | vine stem | (1)Tonic for debilited people with pale skin complexion, (2) difficult urination | Stem decoction used to give appetite and as a tonic. (2) Stem decocotion drank all day long to clean the kidneys. |
| JMD 733 | *Uncaria macrophylla* Wall. | Rubiaceae | pos kuab yag ntsuab | root | Amenorrhea | A large thick root is placed in water and boiled untill the color change. This preparation is drunk all day long for 6 days. |
| JMD 348 | *Uncaria scandens* (Sm.) Hutch. | Rubiaceae | pos kub yab | root | Male sexual weakness | Root decoction drunk all day. |
| JMD 106 | *Urena lobata* L. | Malvaceae | tsim mos tsab liab / nrhab hawj sam | root | Vaginitis, local vaginal inflamation | Root decoction drank warm (3 times a day). |
| JMD 721 | *Urena lobata* L. | Malvaceae | nrhab los yaj | root and stem | Leucorrhoea with pelvic pain | *Evodia* sp.719- *Hedyotis capitellata*  Wall. ex G.Don 720-*Urena lobata* L. 721 *Cheilocostus speciosus* (J. Koenig) C.D. 722 Specht -*Mussaenda* *pubescens* W.T. Aiton 723-*Clerodendrum schmidtii* C.B.Clarke 724. are prepared in decoction (until the color changes) |
| JMD 323 | *Usnea* sp. | Parmeliaceae | ntx huab ntoos | whole plant | Painfull white spot in mouth, mouth ulcera, aphta | A decoction of the whole plant is used as a gargle 3-4 times a day. Don't swallow. |
| JMD 336 bis | *Verbena officinalis* L. | Verbenaceae | tshuaj zoo qoob | leaf | Dermatitis (red spots) wih fever | Crushed leaves are prepared in infusion (10 min), to drink. |
| EE55 | *Vernicia montana* Lour. | Euphorbiaceae | txiv thooj ywg | fruit with seed | Pain in testicles | as tu la recette ? |
| JMD 703 | *Vernonia* sp. | Compositae | ntiv | leaf | (1) Sprains, broken bones (2) for post partum period, to ease placenta delivery | (1) Crushed leaves of *Sambuccus javanica* (600) and Vernonia sp.( 703) and applied as a poultice on affected area.  (2) To ease placenta delivery: leaves of *Sambuccus javanica* (600) and *Vernonia* sp.(703) are chopped and cooked in chicken broth. |
| JMD 314 | *Viburnum cylindricum* Buch.-Ham. ex D. Don | Caprifoliaceae | txiv ntxia zoov | leaf and root and stem | Dryness in mouth and throat, dry cough with fever | Leaves, root and wood are prepared in decoction (10 min) to drink all day. |
| JMD 359 | Viburnum cylindricum Buch.-Ham. ex D. Don | Caprifoliaceae | ntoo quav qa | aerial part | Food poisoning (with vomiting) | Bark, wood, leaves are prepared in form of a decoction whih is drunk all day long. |
| JMD 319 | *Viscum articulatum* Burm. f. | Viscaceae | hmab tsob ntoos | whole plant | Broken bone | Crush and use in poultice around broken bone area. |
| JMD 122 | *Volkameria inermis* L. | Verbenaceae | ntshaub rws liab | root | Blood in feces | Chopped root are prepared in decoction which is drunk. |
| JMD 861 | *Wendlandia* sp. | Rubiaceae | ntoo liab | stem and root | Ovarian cyst | *Mallotus barbatus* Müll. Arg (860), *Wendlandia* sp.( 861) and indet. (862) are prepared in docction and drunk until cured. |
| JMD 316 | *Yua thomsonii* (M.A. Lawson) C.L. Li | Vitaceae | hmab tsob xyoob | whole plant | Haematoma | The whole plant is crushed and applied on the bruise. |
| JMD 329 | *Zanthoxylum acanthopodium* DC. | Rutaceae | txiv xiav | sap | Skin outbreak (red spots) with fever | Pieces of wood are placed on amber. The leaking sap is collected in a glass and drunk. |
| JMD 308 | *Zephyranthes* sp. | Amaryllidaceae | txawb ceeb | whole plant | Bleeding wound | Crush the whole plant and apply in poultice on the wound. It is a very efficient remedy. |
| JMD 258 | *Zingiber zerumbet* (L.) Roscoe ex Sm. | Zingiberaceae | qoov nkag | leaf and stem | Internal hemorrhage | Leaves and stem are cooked in form of chicken broth and eaten. |
| JMD 857 | *Ziziphus attopoensis* Pierre | Rhamnaceae | hmab pos | stem | Male sexual weakness | The stem of *Ziziphus attopoensis* Pierre (857) is mixed with *Sterculia* *lanceolata*Cav. (858) and prepared in form of a decoction which is drunk. |
| JMD 79 | *Ziziphus oenopolia* (L.) Mill. | Rhamnaceae | hmab pos rau tshi soob | root | Sprains, traumas in ligaments and tendons, tendinitis | Root decoction is drunk. |
